# Finite particle size drives defect-mediated domain structures in strongly confined colloidal liquid crystals

**DOI:** 10.1038/ncomms12112

**Published:** 2016-06-29

**Authors:** Ioana C. Gârlea, Pieter Mulder, José Alvarado, Oliver Dammone, Dirk G. A. L. Aarts, M. Pavlik Lettinga, Gijsje H. Koenderink, Bela M. Mulder

**Affiliations:** 1 Department of Systems Biophysics, FOM Institute AMOLF, Science Park 104, 1098XG Amsterdam, The Netherlands; 2 Department of Chemistry, Physical and Theoretical Chemistry Laboratory, University of Oxford, South Parks Road, Oxford OX1 3QZ, UK; 3 Institute of Complex Systems (ICS-3), Forschungszentrum Jülich, 52425 Jülich, Germany

## Abstract

When liquid crystals are confined to finite volumes, the competition between the surface anchoring imposed by the boundaries and the intrinsic orientational symmetry-breaking of these materials gives rise to a host of intriguing phenomena involving topological defect structures. For synthetic molecular mesogens, like the ones used in liquid-crystal displays, these defect structures are independent of the size of the molecules and well described by continuum theories. In contrast, colloidal systems such as carbon nanotubes and biopolymers have micron-sized lengths, so continuum descriptions are expected to break down under strong confinement conditions. Here, we show, by a combination of computer simulations and experiments with virus particles in tailor-made disk- and annulus-shaped microchambers, that strong confinement of colloidal liquid crystals leads to novel defect-stabilized symmetrical domain structures. These finite-size effects point to a potential for designing optically active microstructures, exploiting the as yet unexplored regime of highly confined liquid crystals.

## Abstract (web summary)

Liquid crystals confined to micrometre-sized geometries can be well described by a continuum theory, where the size effect of constituent mesogens is negligible. Here, the authors show how the continuum theory breaks down in colloidal liquid crystal, leading to the formation of defect-mediated domains.

## Graphical abstract

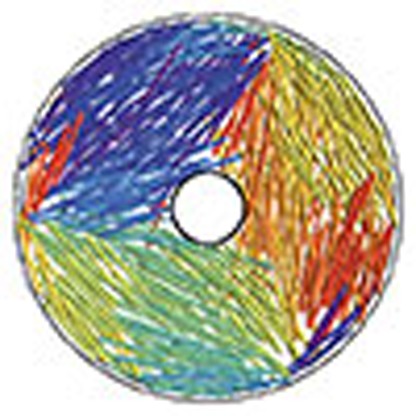

The study of liquid crystals in confinement has a history stretching back to the days of the development of the first twisted nematic display cells^1^. Confined liquid crystals attracted the attention of fundamental scientists, as they provided a window on a host of intriguing phenomena.

First and foremost, this concerned the study of topological defects, which in these systems arise both spontaneously or can be induced and modulated through the geometry of confinement and the imposed boundary conditions. Starting in the 1970s a major effort was undertaken to understand and classify these defect structures^2^,^3^,^4^,^5^. In the 1980s the focus was on spherical confinement geometry^5^,^6^, spurred in part by the development of polymer dispersed liquid-crystal displays^7^. Later on, it was realized that colloidal particles, suspended in a liquid-crystal and surface-treated to impose specific orientational anchoring conditions, create defect structures that have a major role in mediating the interactions between these particles^8^,^9^,^10^,^11^,^12^. An overview of this field can be found in the comprehensive review by Kleman and Lavrentovich^13^. Interest in this area has remained unabated, and more recent work has covered effects such as frustration^14^, complex defect dynamics^15^,^16^, bistability^17^ and tuneable interactions between colloids^18^,^19^. However, a common element in all this work was the fact that the size of the anisotropic particles involved was irrelevant. Indeed, standard thermotropic mesogens typically have lengths below 2 nm, and are therefore negligible in size even in micrometer-sized confining volumes or compared to the diameter of typical colloidal solutes. The physics of these systems is therefore fully captured by continuum theories^20^, which describe the local anisotropy of the material by vector or tensor fields that are continuous, except possibly at isolated points, lines or surfaces where defects are localized. Arguably, in some cases the nematic coherence length^21^, which governs the spatial extent by which boundary conditions influence the bulk behaviour, can reach dimensions comparable to the system size. However, it is an open question what happens when the size of the particles themselves does become important.

In order to reach this regime, we take a cue from nature, which provides a host of viral particles and filamentous biopolymers whose lengths are upward of the micrometer scale. Moreover, biopolymers in nature are actually often strongly confined. Cytoskeletal filaments for instance are packed at high density within thin sheet-like lamellipodia and finger-like filopodia^22^ and viral DNA is packed at high density inside tiny virus capsules^23^. These strongly confined conditions in which the polymer length is comparable to the typical dimensions of the confining space raise the general question how colloidal nematics resolve the topological and geometric constraints that inevitably arise when mutual packing and alignment to boundaries compete at the same length scale.

To address this question in a well-controlled setting, we will consider model systems composed of rod-like particles, using both simulations and experiments with *fd*-virus particles.

Since the interactions between colloidal particles, especially in physiological salt conditions, are dominated by short-range repulsions, the interactions between the particles in the simulations are taken to be of the hard body type. Since the seminal work of Onsager^24^, it is known that such systems display entropy-driven liquid crystalline phases. The bulk phase diagram of hard rods has been studied extensively by computer simulations^25^. Several groups have shown that colloidal systems composed of rod-like virus particles are indeed well described by this model (for a review see ref. 26). There are few studies that have looked into the effect of confinement in a single direction^27^,^28^, but until recently studies with full confinement in all dimensions were scarce^29^,^30^,^31^. We focus on quasi 2-dimensional (2D), circularly symmetric confinement geometries, ideally suited for analysis and visualization purposes, and readily adapted to probe different confinement topologies. In the following, we first report on the simulations, followed by the experimental results. Our main result is that, depending on the geometry and the topology of the confining volume, and the relative size and packing fraction of the particles, a wide variety of defect-mediated patterns emerge. For a circular geometry these patterns are characterized by antipodal pairs of isolated singularities, which are either located within the volume or (virtually) outside it. For the topologically distinct annular geometry, *n*-fold symmetric domain structures appear, controlled by the size of the inner hole.

## Results

### Circular geometry

**Figure 1:**
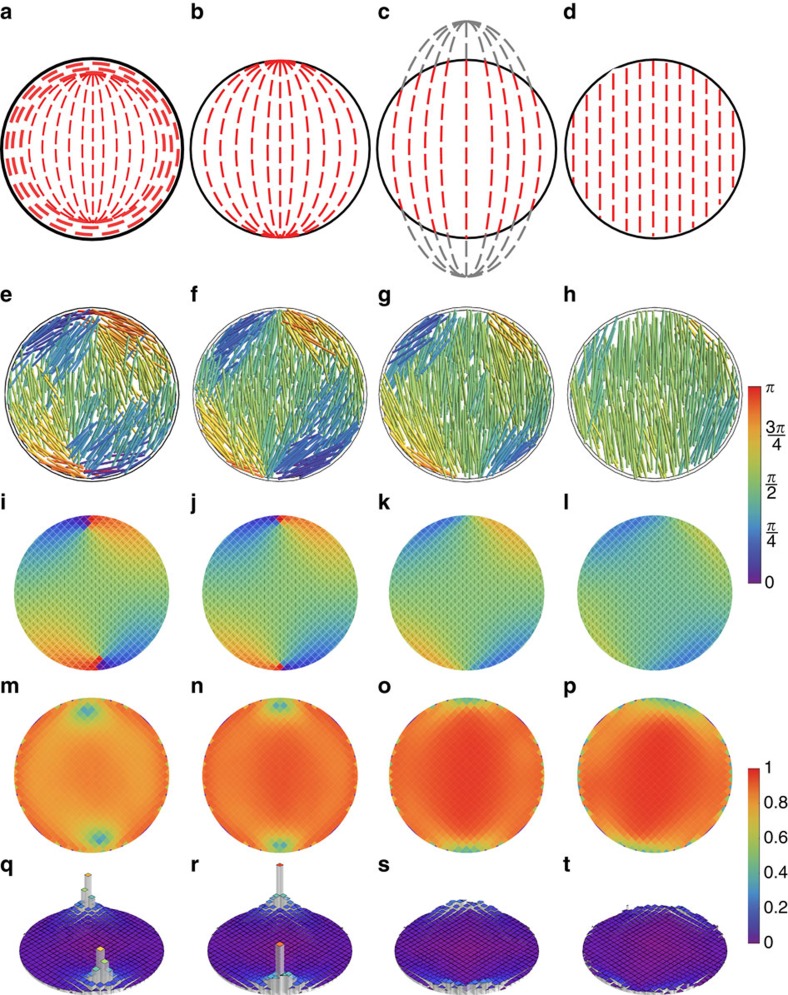
Patterns of rod-like particles in circular confinement. Overview of packing structures of rod-like particles confined in shallow chambers with a circular geometry. Top row: schematic showing classification by the location of the disclination points: (**a**) type *B*_i_, (**b**) type *B*_b_, (**c**) type *B*_o_, (**d**) type *B*_∞_. Second row: snapshots showing particle positions and their orientations with respect to the vertical (mod *π*) (see colour bar on the right). Third row: orientation patterns averaged over >10^3^ configurations. Fourth row: value of the scalar order parameter *S*∈[0,1], scale bar on the right. For a discussion of the error estimate in this quantity see the Supplementary Note 8 and Supplementary Fig. 10 Note the characteristic dips at the location of the defects. Fifth row: normalized angular deficit parameter, which peaks at the centre of the defects. Simulation parameters: (**e**,**i**,**m**,**q**) *L*/*D*=15 and *η*=0.16; (**f**,**j**,**n**,**r**) *L*/*D*=15 and *η*=0.20; (**g**,**k**,**o**,**s**) *L*/*D*=20 and *η*=0.20; (**h**,**l**,**p**,**t**) *L*/*D*=25 and *η*=0.20.

As a canonical baseline model, we first consider a simple circular geometry. For details on the simulation technique and the simulation geometry we refer to the Methods section. As shown in Fig. 1, we observe a variety of packing structures that are all controlled by two in-plane singularities of strength +1/2. These singularities are in fact the end-points of line defects that span the system from top to bottom, but due to dominant planarization effect of the top and bottom boundaries, the system is effectively homogeneous in this direction and out of plane orientations are negligible. We therefore proceed to characterize them by their in-plane nature. A 2D disk has Euler characteristic *χ*=1 (ref. 32), which dictates that any orientational pattern aligned to the perimeter should carry a topological charge of +1 (ref. 33). Indeed, a 2D version of continuum theory applied to the case with strong parallel boundary anchoring predicts a bipolar pattern consisting of two diametrically opposite +1/2 singularities located at the perimeter of the disc as the stable state (Supplementary Note 1; Supplementary Fig. 1). Although these simulations employ the 2D tensor order parameter^34^, due to the observed strong degree of planarization the 3D order parameter we measure in our simulations effectively coincides with the 2D one (Supplementary Note 2). For a full discussion on our characterization of the singularities please refer to Supplementary Note 3.

Depending on the aspect ratio and the packing fraction of the particles, we find that the singularities are located inside the disc (*B*_i_), at its boundary (*B*_b_) or are present as virtual singularities located either a finite distance from the centre (*B*_o_), but still causing a noticeable distortion of the alignment pattern on the inside or effectively infinitely far away (*B*_∞_), yielding a nearly homogeneous nematic state. The virtually bipolar patterns (*B*_o_ and *B*_∞_) have been predicted to occur under weak anchoring conditions in elongated droplet shapes^35^,^36^, and reflect the fact that the entropy-driven alignment of the particles to the in-plane boundaries can at higher densities be overridden by the bulk alignment. The *B*_i_ pattern observed for shorter rods and at relatively low density in which a finite boundary layer of wall-aligned particles encloses the two singularities, however, was hitherto not described, and provides a clear signal that local packing effects can cause deviations from the continuum picture. An overview of the patterns found in the circular geometry can be found in Supplementary Table 1. Our results demonstrate that the patterns observed in strictly 2D systems^37^,^38^,^39^ are robust against the presence of a finite system size in the transverse direction^30^.

### Annular geometry

**Figure 2:**
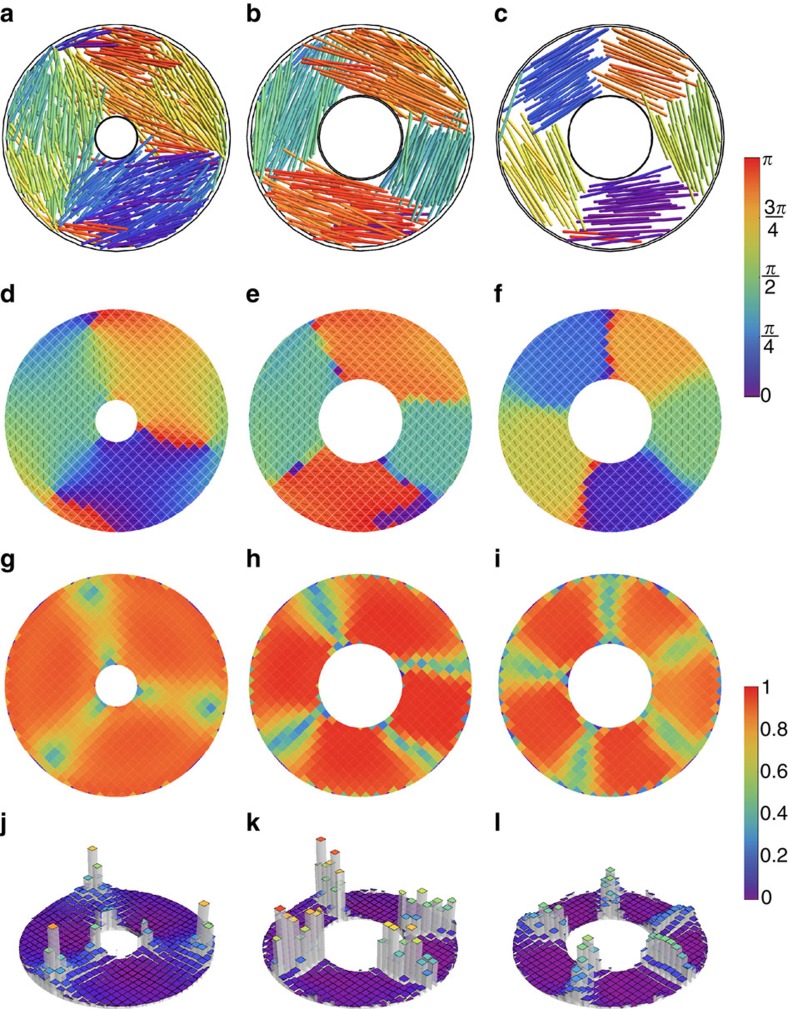
Patterns of rod-like particles in annular confinement. Overview of packing structures of rod-like particles confined in annulus-shaped chambers, showing threefold (left column), fourfold (middle column) and fivefold (right column) symmetry. Top row: snapshots of simulations. Second row: particle orientations averaged over averaged over >10^3^ configurations, labelled by colour bar on the right. Third row: scalar order parameter *S*∈[0,1]. Fourth row: normalized angular deficit parameter. Simulation parameters for each column (from left to right): (**a**,**d**,**g**,**j**) *H*=6, *L*/*D*=15, *η*=0.20 and *R*_inner_=7.5; (**b**,**e**,**h**,**k**) *H*=6, *L*/*D*=25, *η*=0.20 and *R*_inner_=15; (**c**,**f**,**i**,**l**) *H*=3, *L*/*D*=25, *η*=0.20 and *R*_inner_=15. For error calculations see Supplementary Note 8 and Supplementary Fig. 10.

What happens when we change the topology to that of a 2D annulus? This shape has Euler characteristic *χ*=0, which suggests that singularities can be avoided. Indeed, the continuum theory of 2D liquid crystals with strong parallel anchoring predicts that in this case a defect-free, tangentially ordered nematic structure is stable (Supplementary Note 1; Supplementary Fig. 1). To test whether this prediction still holds for the finite particle size, we open a hole with a varying inner radius *R*_inner_ in the centre of our simulation volume. Strikingly, we see that with increasing *R*_inner_, different *n*-fold symmetric domain structures develop (Fig. 2), which we denote by the dihedral group symbol D_*n*_ (ref. 40). For the case D_3_, we find domains with a boundary that is demarcated by a+1/2 singularity located at the outer rim and a −1/2 singularity at the inner rim. Inspection of snapshots of the simulations reveals that in this case, a particle can fit radially inside the container and form a bridge between the two singularities. For the patterns with *n*≥4 this is no longer possible, and the boundary between the domains is an extended disclination wall. Interestingly, a recent simulation study reported the D_3_ pattern as a metastable state for the 2D circular system^41^, possibly an effect of specific initial conditions. In our simulations, however, we have never observed this pattern in the circular system and we argue that it is only stable in the annular system. An overview of the patterns found in the annular geometry can be found in Supplementary Table 2.

The discrete nature of the domain structure appears to be dominated by the possible packing arrangements of the rods around a central hole of a given size. Considering the inner hole radii that would perfectly inscribe a regular *n*-gon of particles, one obtains the relation 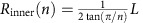.

**Figure 3:**
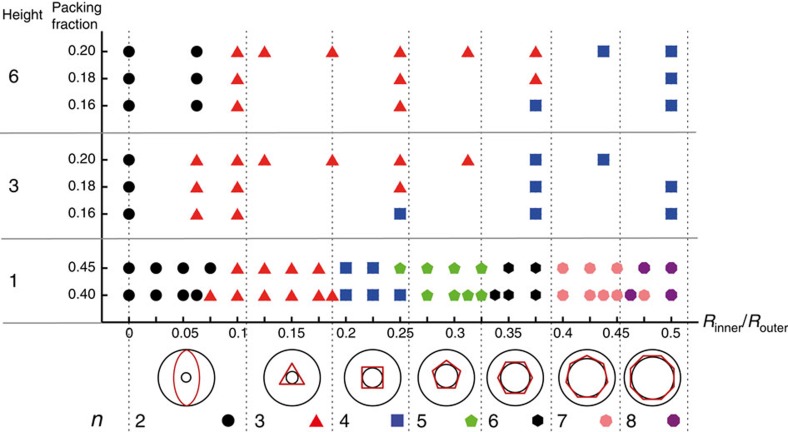
Number of nematic domains in different geometries. The symmetry number *n* of the patterns observed in simulations as a function of the inner hole radius *R*_inner_ for rods with aspect ratio *L*/*D*=15 in an annular geometry with *R*_outer_=40*D*, for chamber heights *H*=*D* (the strictly 2D case) and *H*=3*D*, 6*D* at a number of different packing fractions. Each coloured symbol represents a different pattern, the chosen shape indicating the symmetry as explained in the legend at the bottom. The dotted vertical lines mark the predicted boundaries between the different patterns based on the geometrical rule discussed in the main text.

Pooling all our simulation data on the observed patterns in Fig. 3, shows that indeed this geometrical criterion almost exactly matches the observed boundaries between the different regimes for *H*=*D* (Supplementary Fig. 2), while the ranges of stability of D_3_ and D_4_ patterns widen significantly for the systems with *H*=3*D* and 6*D*.

### Experiments

The fact that the simulations indicate that the D_3_ pattern is stable for a wider range of geometries when the height of the well increases, suggests that this pattern should be observable even when the extreme transverse confinement conditions of the simulation are not fully met. We experimentally realized such a system by confining nematic liquid crystals of bacteriophage *fd*-virus rods^42^ inside shallow, annulus-shaped microchambers. The *fd*-virus particles are the convenient model liquid-crystal system, since they are monodisperse in length, their interactions are hard-core like when surface charges are screened and their bulk phase behaviour is well-known^43^. *fd*-rods have a length of *L*=0.88 μm and diameter of *D*=6.6 nm, and we can observe individual particles and their anisotropic (mostly axial) diffusion in the nematic background by fluorescence microscopy^44^. We use photolithography to produce non-adhesive annulus-shaped microchambers whose outer radius *R*_outer_ ranges from 5 to 50 μm, yielding confinement in the range *κ*=10^−1^–10^−2^, and whose inner hole radius *R*_inner_ ranges from 0 to 0.7 times the outer radius *R*_outer_. The chambers have a height in the range of *H*=1–3 μm, which is the minimal thickness that was experimentally attainable. We acquired confocal fluorescence time-lapse image series of chambers containing *fd*-rods with a small fraction of fluorescently labelled rods serving as tracers. To compute director fields, we extract the time-averaged orientation of the nematic director for each image pixel by the automated image analysis (Supplementary Note 4; Supplementary Figs 3 and 4).

**Figure 4:**
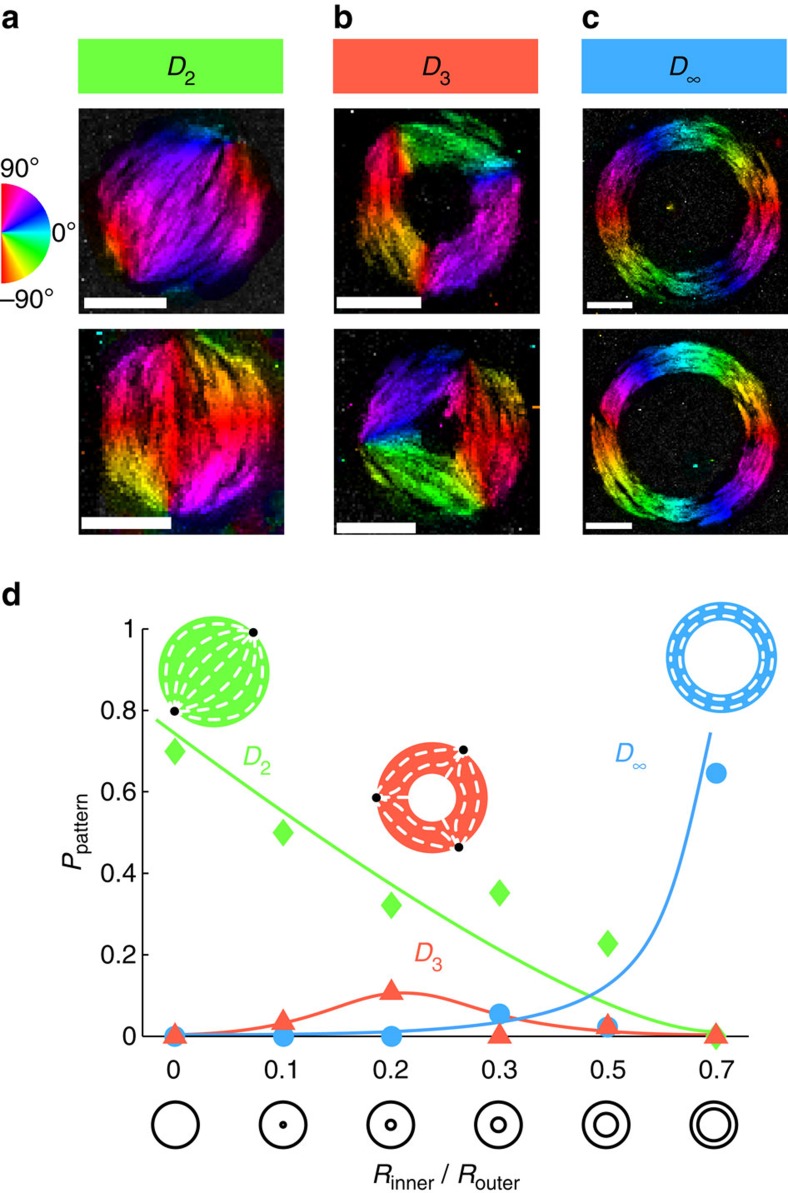
Experimentally observed patterns. Structures observed in colloidal liquid crystals confined to disk-shaped and annulus-shaped microchambers. (**a**–**c**) Representative images of three types of director-field patterns. Hue corresponds to average orientation (Supplementary Note 4; Supplementary Figs 3 and 4) according to legend (left). Brightness corresponds to a maximum-time projection over 2,000 acquired frames of fluorescently labelled *fd*-virus particles. (**a**) *D*_2_: director field exhibits twofold symmetry and two singularities. (**b**) *D*_3_: director field exhibits threefold symmetry and three singularities. (**c**) *D*_∞_: director field exhibits full rotational symmetry and no singularities. Scale bars, 5 μm. (**d**) Probability *P*_pattern_ that a given pattern occurs as a function of inner diameter *R*_inner_ (in units of outer diameter *R*_outer_). *D*_2_ (green diamonds) is most likely for *R*_inner_=0; *D*_3_ (red triangles) is most likely for *R*_inner_/*R*_outer_=0.2; *D*_∞_ (blue circles) is most likely for *R*_inner_/*R*_outer_=0.7. Lines are guides to the eye. Other patterns were also found (Supplementary Note 5; Supplementary Figs 5 and 6).

We observe mainly three distinct patterns: a twofold symmetric structure (*D*_2_) showing two opposite +1/2 singularities (Fig. 4a), a threefold symmetric pattern (*D*_3_) showing three +1/2 singularities (Fig. 4b) and a pattern with infinite-fold rotational symmetry (*D*_∞_) that lacks singularities (Fig. 4c). The occurrence of these patterns strongly depends on the shape and size of the confining chambers, as shown in Fig. 4d. Strikingly, the *D*_2_ pattern, which corresponds to the *B*_b_ pattern predicted by simulations of rods in a disk-like geometry (Fig. 1b) and in an annular geometry with small inner hole radius (Fig. 3), persists over a wide range of inner hole radii (Fig. 4d). The *D*_3_ pattern only occurs for chambers with a small but finite hole size in the range of 1–3 μm, corresponding to ∼1–3 *fd*-rod lengths and *R*_inner_/*R*_outer_=0.2 (Fig. 4d, red triangles). This pattern has three prominently visible and evenly spaced +1/2 singularities. Finally, the pattern with infinite-fold symmetry dominates for systems with a large inner hole (*R*_inner_/*R*_outer_=0.7, Fig. 4d, blue circles). The experiments are consistent with the simulation results, notably the extended stability range of the *D*_3_ pattern in chambers of finite height. Unfortunately, we did not observe the *D*_*n*≥4_ patterns, but note that the *D*_∞_ pattern is the logical limit of the patterns with increasing finite *n*-fold symmetry predicted for increasing inner hole radius. Remarkably, in spite of the fact that the experimentally realized heights in principle allow the *fd*-particles to fully rotate out of plane, the observed patterns are completely planar. This suggests that the entropically favourable planar degenerate boundary conditions imposed by the large top and bottom surfaces in conjunction with the long-ranged orientational order in the nematic state are sufficient to impose quasi-2D behaviour for a large range of box geometries. A full overview of the patterns found in the experiments is given in Supplementary Note 5 and Supplementary Figs 5 and 6.

## Discussion

Our results show that strongly confined colloidal liquid crystals have a rich phase behaviour mediated by defect structures. These intricate coupled spatial and orientational patterns arise from the complex interplay between particle size and shape, and the geometry and topology of the confinement volume. The description of these effects is beyond the reach of standard continuum theories, which neglect finite particle size effects and require singularities to be incorporated ‘by hand'. This raises the challenge of developing a tractable theoretical framework in which the ratio of the particle dimension to the characteristic length scale(s) is a salient parameter. However, our results show that simulations, albeit on systems that are as yet significantly smaller than the experimentally realized ones (for a critical comparison between the two please see Supplementary Note 6 and Supplementary Fig. 7), already provide predictive insights. Finally, our findings open a novel avenue to create liquid-crystal systems with designed orientational microdomain structures^45^, with the potential for creating controlled optical properties.

## Methods

### Simulations

We use the standard Metropolis Monte Carlo technique to simulate hard particles, sampling both rotations and translations of the particles, and accepting these if no overlaps with other particles or with the confining walls are created^46^. As model particles, we consider hard, rigid spherocylindrical rods, of length *L* and diameter *D*. These particles are confined in shallow, circular microchambers with radius *R*_outer_. Two parallel plates with spacing H smaller than the particle length formed the top and bottom of our simulation volumes, allowing us to focus on in-plane pattern formation.

The excluded volume interactions of the rods with the walls favour planar degenerate boundary conditions^21^ with particles aligned parallel to the boundaries. Note, however, that, because of the finite size of the particles and the finite radius of curvature of the side walls, the centre of mass of the particles is constrained to keep a distance >*D* from the side walls. The alignment with the lateral boundaries is therefore at best approximate. We considered both the strictly 2D case in which the height is equal to the diameter of the rods (*H*=*D*), as well as more realistic quasi-2D situations, *H*=3*D* and 6*D*. We want to study the regime in which the interparticle alignment competes with the boundary alignment. Since the boundary anchoring length for spherocylindrical rods is of the order of the particle length,^27^,^47^ we need to choose *R*_outer_ to be comparable to *L*. A convenient measure for the degree of lateral confinement is the ratio *κ*=*L*/(2*R*_outer_), which is unity when the rods can just fit in the volume and zero for the unconfined case. In order to obtain a reasonable compromise between computational tractability and realism we used an outer radius *R*_outer_=40*D* and particle aspect ratios in the range *L*/*D*=15–25, yielding *κ*=0.2–0.3. The number of particles was chosen to obtain packing fractions at which the corresponding unconfined system is in a nematic state^25^. For the circular geometry this implies that we have between 300–500 particles for *H*=6*D*. To systematically characterize any patterns observed, we measured a spatially resolved version of the standard second-rank tensorial order parameter. Local order parameter tensors have already been employed previously in the study of inhomogeneous liquid crystals, both in lattice models^48^ and even earlier in off-lattice models^49^. In these studies, the spatial variations were on a scale small with respect to the system size, but typically larger than the size of the unit cell (for the lattice models) or the particle size (in the off-lattice case). Here, however, we are using an order parameter, which is defined at a length scale smaller than that of the particles, and which also varies on this length scale. Such an order parameter has in fact already been used extensively in the study of liquid crystalline polymers (see for example, ref. 50), and has recently been shown to consistently carry over to rigid particles^51^,^52^. For more information on the order parameter, please refer to Supplementary Note 2 and Supplementary Fig. 8. The order parameter allows us to extract the average local degree of order, the local preferred axis of ordering, and the local angular deficit, which is a sensitive measure for the presence of orientational singularities (Supplementary Note 7). It should be noted that these singularities are in the orientation field, and are not accompanied by any significant change in the local packing fraction of the rods (Supplementary Fig. 9). To monitor the equilibration of our system, we use the largest positive eigenvalue of the global tensor order parameter as a scalar measure of ordering. To analyze the statistical reliability of our results we have performed an error analysis using subsamples, which is described in Supplementary Note 8 and Supplementary Fig. 10.

### Bacteriophage *fd*-virus preparation

*fd*-virus rods were grown using a standard protocol^26^ and stored in *fd*-buffer solution (20 mM tris, pH 8.15, 100 mM sodium chloride, 15% ethanol). Assay suspensions were prepared at concentrations of 24 mg ml^−1^, slightly above the bulk isotropic–nematic biphasic region, which occurs at ∼20 mg ml^−1^, in agreement with the Onsager theory^53^. Bulk suspensions were biphasic, as evidenced by visual inspection through crossed polarizers. We chose the lowest possible nematic concentration, in order to minimize the energy cost to re-arrange from one director field to another and thus reduce the probability of getting stuck in high-energy metastable states. Fluorescently labelled rods were prepared by incubation with Alexa-488 succinimidyl ester (Invitrogen) following a published procedure^47^. A small fraction of labelled rods (2–4% v/v) was mixed with unlabelled rods in order to make individual-labelled rods distinguishable by fluorescence microscopy.

### Microchamber preparation

Microchambers were assembled using a standard photolithographic technique described elsewhere^30^. Chamber dimensions were set by a mask design with circular and annular geometries with outer radii of 5, 10, 15, 25, 35 and 50 μm. For each outer radius, geometries with inner radii of 0, 0.1, 0.2, 0.3, 0.5 and 0.7 times the outer radius were made, resulting in a total of 36 different geometries. Chambers were sealed with polydimethylsiloxane-coated microscope slides and soaked overnight in *fd*-buffer containing 0.1 wt% of the amphiphilic block copolymer Pluronic F-127 (Sigma-Aldrich). This treatment effectively blocked nonspecific adsorption of *fd*-rods, as confirmed by time-lapse imaging of rod diffusion. Saturation of the PDMS with buffer prevented drying of the sample for at least 24 h.

### Confinement assay

A drop of *fd*-virus suspensions was placed on a glass-photoresist substrate and pressed against rubber-coated glass to form microchambers. We hermetically sealed the glass edges with VALAP and let samples equilibrate for at least 30 min before visualizing by fluorescence microscopy. We only consider well-sealed chambers for quantification. Approximately 40% of all chambers are well-sealed. Chambers that were not well-sealed were evident by fluorescently labelled rods escaping from the chamber. The rods rapidly organized in steady-state nematic patterns within 30 min after filling the chambers. When we filled the chambers with the isotropic phase below the biphasic region, a nematic was not formed and the samples remained isotropic. This observation implies that the filling (and confinement) were not sufficient to induce a nematic.

### Fluorescence microscopy

Microchambers were visualized using two microscope setups: (1) a spinning disk confocal scanner (CSU 22, Yokogawa) on an inverted microscope (DMIRB, Leica) with a cooled, electron-multiplying charged-coupled device (C9100, Hamamatsu) and (2) a Nikon C1 confocal point scanner on an inverted microscope (Ti, Nikon) with a photomultiplier tube detector (A1, Nikon). Labelled rods were excited with 488 nm laser light (Coherent). A series of images were recorded over a long enough time interval such that viruses diffused across the entire chamber. The average diffusion constant of virus rods in the nematic phase is 1 μm^2^  s^−1^ along the nematic director and 0.1 μm^2 ^s^−1^ perpendicular to the nematic director^44^. These diffusion constants result in diffusion timescales of tens of seconds for diffusion over a distance of one particle length and minutes for diffusing over interparticle distances between fluorescently labelled rods (∼ a few μm). For spinning disk data, typically ten movies of 200 frames each were acquired at a fast imaging rate (0.1 frames per second), which were separated by 2 min to allow rods to diffuse completely across the chamber. For point-scanning confocal data, 15–30 frames were acquired at a slower rate (1 frame per ∼1–2 min) over several fields of view which were automatically acquired and stitched (NIS Elements, Nikon). A customized image analysis technique was developed to determine the average nematic director orientation <*θ>* given time-averaged orientations *θ* of labelled *fd*-rods across all images acquired (Supplementary Note 4; Supplementary Figs 3 and 4).

### Identification of nematic patterns

Rods formed a variety of liquid-crystal patterns. The pattern type was determined by visual inspection of the nematic director fields of all well-filled chambers on the chip. Supplementary Figs 11 and 12 summarize the pattern frequency and probability, respectively, as a function of *R*_outer_ and *R*_inner_/*R*_outer_. We define the probability that a pattern *P* occurs in a given chamber geometry *G* as the number of observed instances of *P* divided by the total number of well-sealed chambers with geometry *G*. A total of 243 chambers were analyzed: 80, 27, 26, 36, 43 and 31 chambers for *R*_inner_/*R*_outer_=0, 0.1, 0.2, 0.3, 0.5 and 0.7, respectively; 77, 75, 55, 25, 9 and 2 chambers for *R*_outer_/μm=5, 10, 15, 25, 35 and 50, respectively; 113, 5, 23, 21, 33, 32 and 16 chambers for patterns *D*_2_, *D*_3_, *D*_∞_, N, A_1_, A_2_, and A_+_, respectively.

### Data availability

The data that support the findings of this study, as well as the computer codes used in the simulations, are available from the corresponding author upon request.

## Additional information

**How to cite this article**: Gârlea, L. C. *et al*. Finite particle size drives defect-mediated domain structures in strongly confined colloidal liquid crystals. *Nat. Commun.* 7:12112 doi: 10.1038/ncomms12112 (2016).

## Supplementary Material

Supplementary Information Supplementary Figures 1-12, Supplementary Tables 1-2, Supplementary Notes 1-8 and Supplementary References
