# Erratum: Finite particle size drives defect-mediated domain structures in strongly confined colloidal liquid crystals

**DOI:** 10.1038/ncomms12495

**Published:** 2016-08-03

**Authors:** Ioana C. Gârlea, Pieter Mulder, José Alvarado, Oliver Dammone, Dirk G. A. L. Aarts, M Pavlik Lettinga, Gijsje H. Koenderink, Bela M. Mulder

*Nature Communications* 7: Article number: 1211210.1038/ncomms12112 (2016); Published: 06 29 2016; Updated: 08 03 2016.

The present address for Ioana C. Gârlea is incorrect in this Article. The correct present address for this author is given below:

Faculty of Physics, University of Vienna, Boltzmanngasse 5, A-1090 Vienna, Austria.

The name of this author is also incorrect in the ‘How to cite this article' section. This section should read:

Gârlea, I. C. *et al.* Finite particle size drives defect-mediated domain structures in strongly confined colloidal liquid crystals. *Nat. Commun.* 7:12112 doi: 10.1038/ncomms12112 (2016).

