# Supplementary material for: Finite particle size drives defect-mediated domain structures in strongly confined colloidal liquid crystals
Source: Nat Commun. 2016 Jun 29;7:12112. doi: 10.1038/ncomms12112 (PMC4931596; doi:10.1038/ncomms12112)
Supplement: Supplementary Information — Supplementary Figures 1-12, Supplementary Tables 1-2, Supplementary Notes 1-8 and Supplementary References [file ncomms12112-s1.pdf]

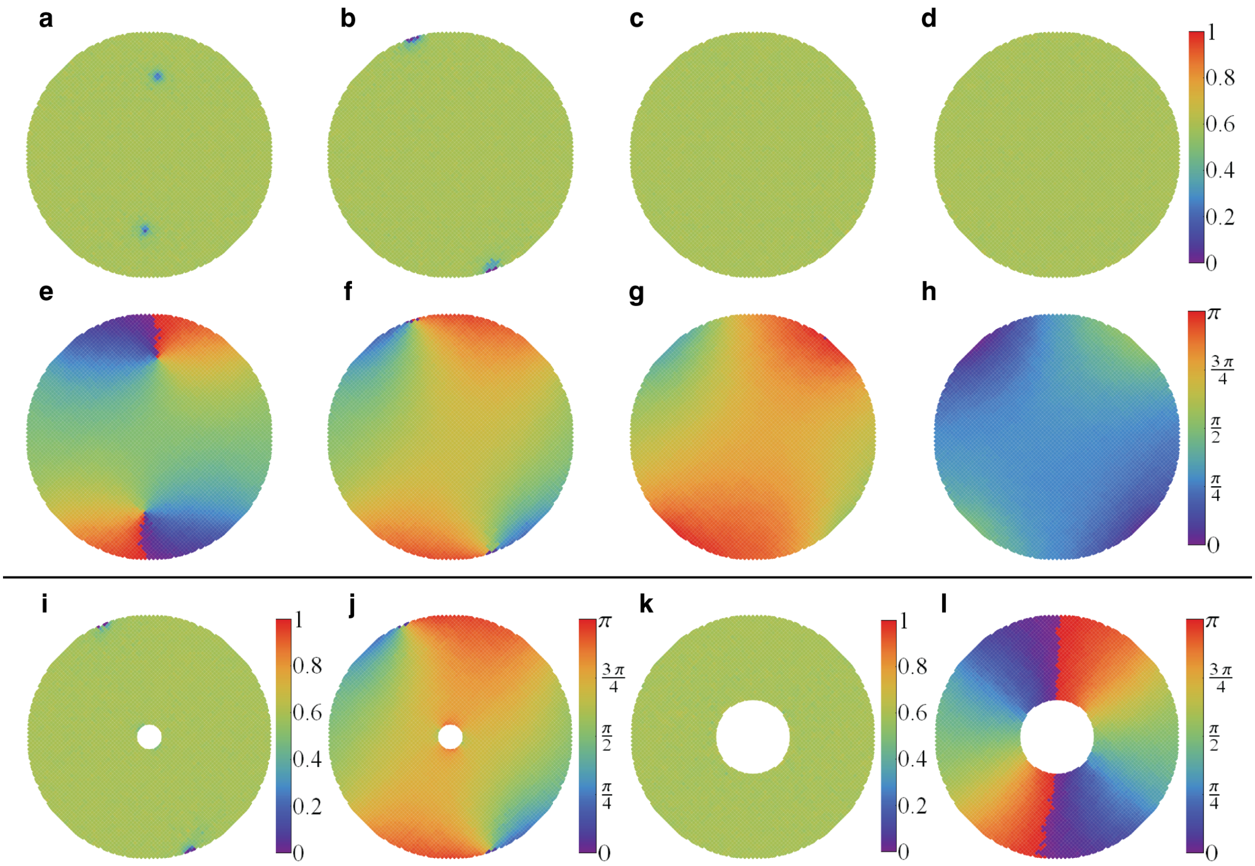

Supplementary Figure 1. Mean-field predictions of orientation patterns for a confined liquid crystal. For panels **a** to **h** a disk-shaped container was considered and for panels **i** to **l** an annulus-shaped container. Panels **a**-**d**, **i** and **k** show the scalar order parameter, while panels **e**-**h**, **j** and **l** show the corresponding orientation. All results were obtained for J=1.25, L_1=0.5 10^{-4} and, L_2=10^{-5}. For the B_i pattern (panels **a** and **e**), W=0.05. For the B_B pattern (panels **b** and **f**) as well as for the two results shown for the annular geometry, W=0.005. The B_O patterns (panels **c** and **g**) were obtained for W=0.5 10^{-3} and the B_∞ patterns (panels **d** and **h**) for W=0.5 10^{-4}. For **i** and **j**, R_{inner} / R_{outer} = 0.1, whereas for **k** and **l**, R_{inner} / R_{outer} = 0.3.

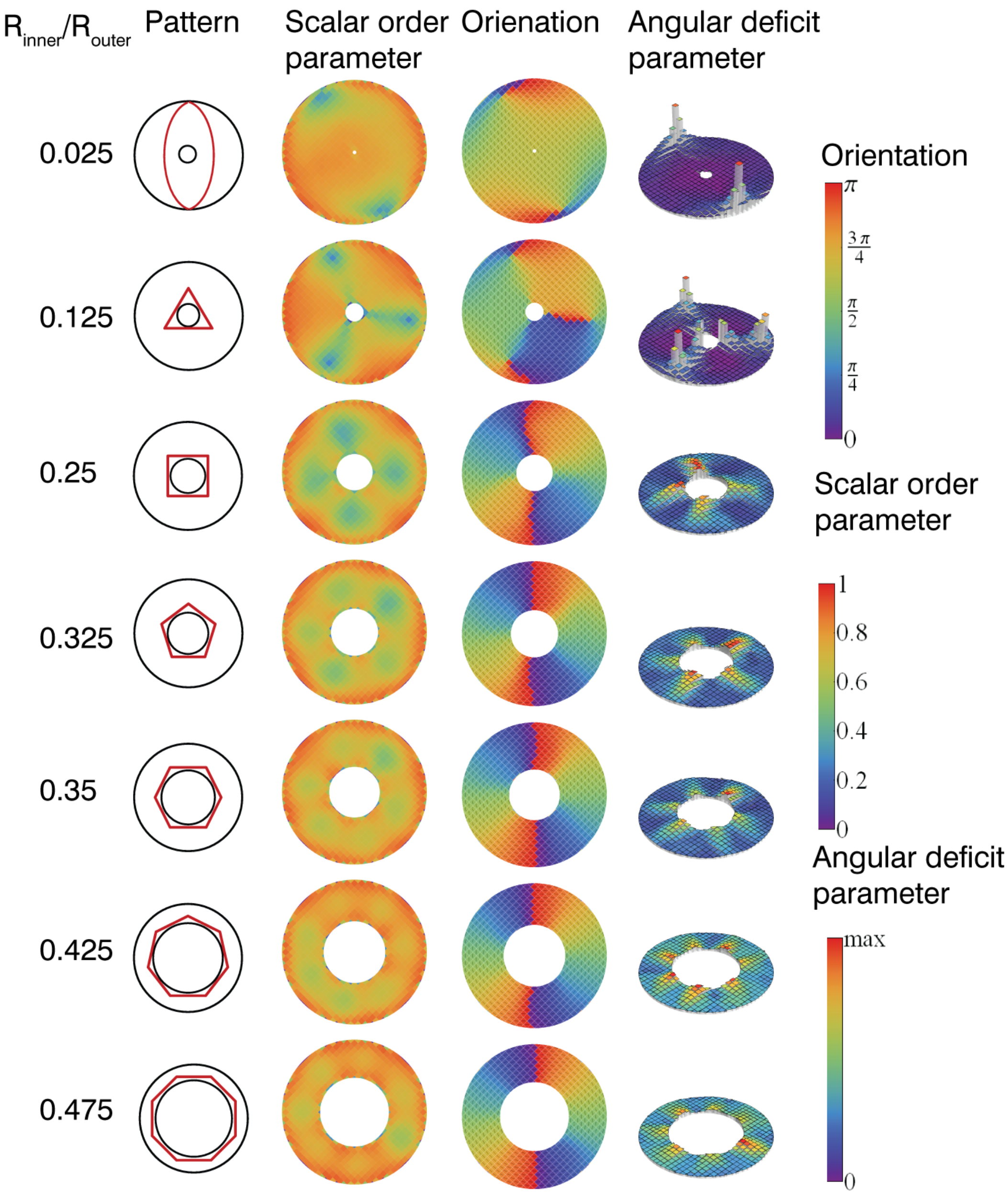

Supplementary Figure 2. Overview of packing structures of rod-like particles confined in 2d annulus-shaped chambers. We observed bipolar structures (top row), 3-fold (second row), 4-fold (third row), 5-fold (forth row), 6-fold (sixth row) , 7-fold (seventh row) and, 8-fold (eighth row) symmetry. Second column: Schematic showing the classification of the patterns. Third column: Particle orientations averaged over 4500 independent configurations, labelled by color bar on the right. Fourth column: Scalar order parameter $S \in [0,1]$. Fifth column: Angular deficit parameter. Simulation parameters: $H = 1, L/D = 15, \eta = 0.40$ and $R_{\text{inner}}/R_{\text{outer}}$ according to first column.

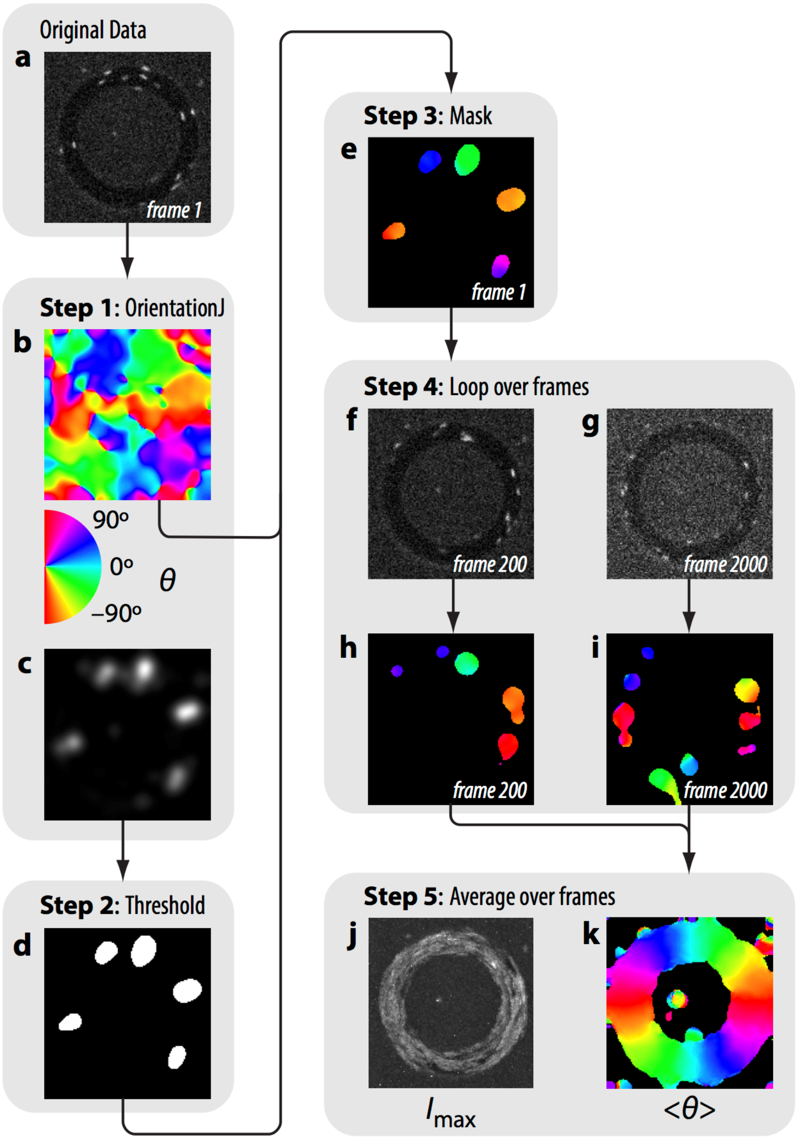

Supplementary Figure 3. Image analysis algorithm. Given many measurements of rod orientation θ for each pixel, the algorithm for determines the average orientation $<\theta>$ of the nematic director. **a**. First frame of original data. **b**. Orientation output of OrientationJ. Color corresponds to orientation θ around each pixel (calibration wheel, below). **c**. Energy output of OrientationJ. Note that energy is highest where changes in fluorescence intensity are largest. **d**. Otsu threshold of the energy image (c). **e**. Masking the orientation image (b) with the threshold image (d). Note that color corresponds to orientation of virus rods in the original image (a). **f–i**. Looping over all frames of a set of images. **f,g**: original data for frames 200 and 2000. **h,i**: Masked orientation images for frames 200 and 2000. **j**: Maximum time projection of the original data for all frames. **k**: Average orientation of all rods, $<\theta>$, which gives the local direction of the nematic director in each image pixel.

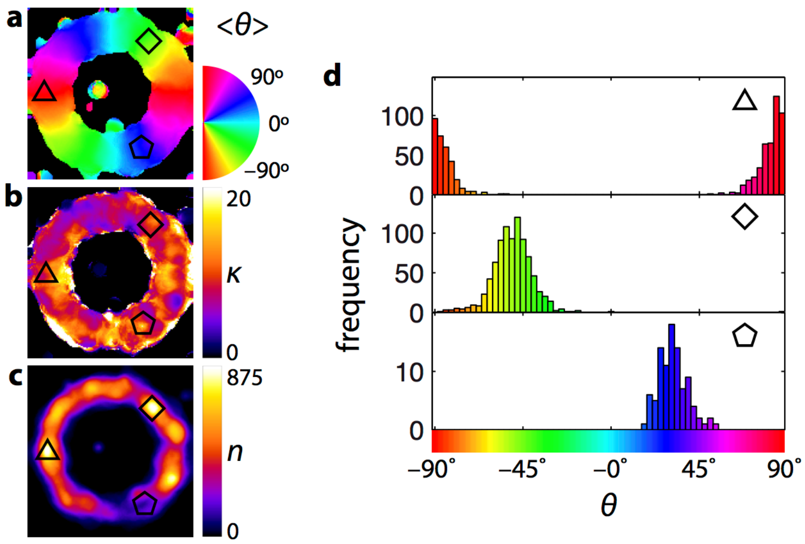

Supplementary Figure 4. Determining mean orientation <θ> from distributions of individual measurements θ. a. Mean orientation <θ>, as in Supplementary Fig. 11, panel k. **b**. Concentration λ for each pixel corresponding to panel a, where color corresponds to values of λ as indicated in the calibration bar on the right. **c**. Number of orientation measurements n per pixel, as determined by the number of frames that a given pixel passes the threshold in Step 2. **d**. Histogram of orientation measurements for the three pixels indicated by triangles, diamonds, and pentagons in panels **a–c**.

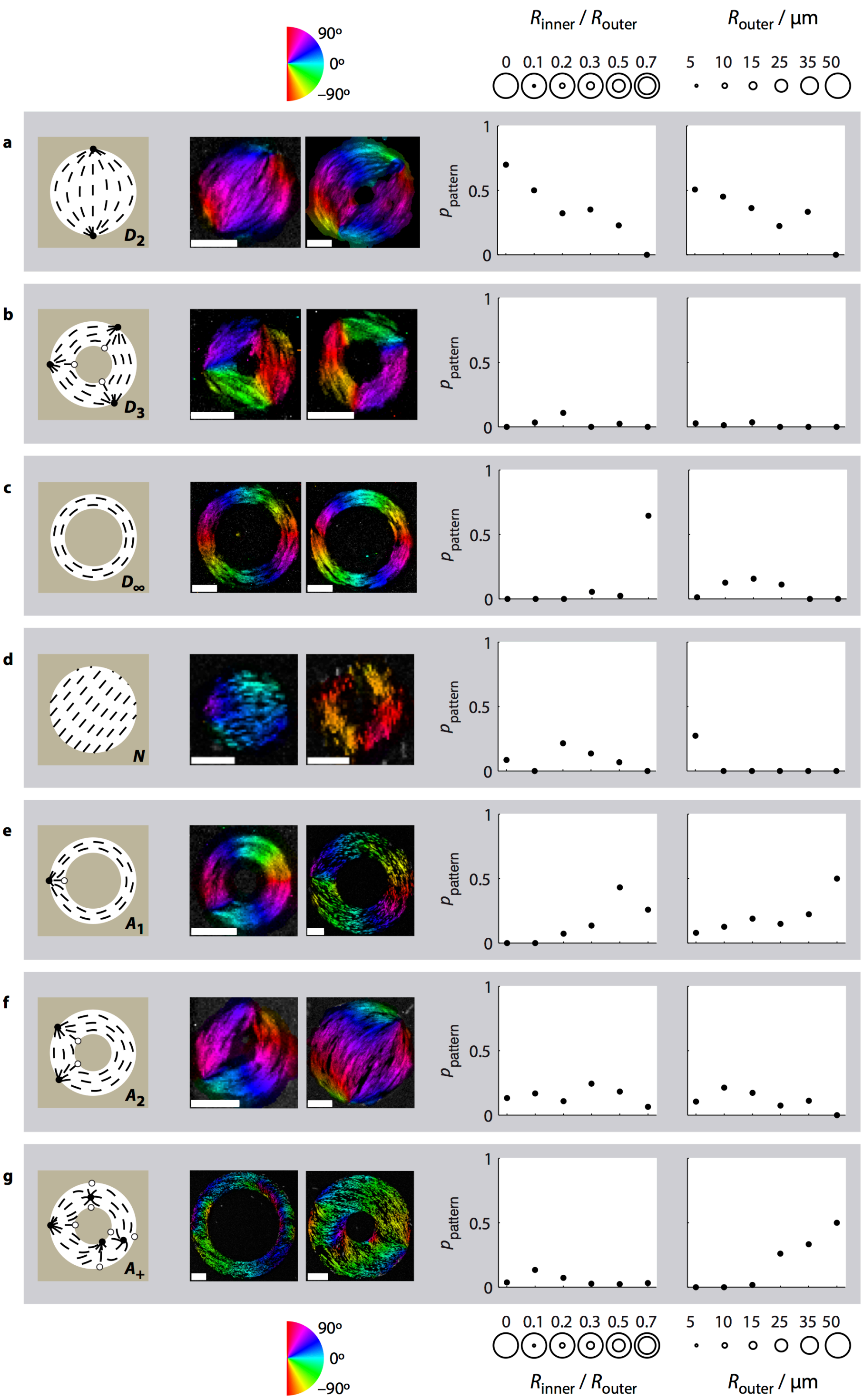

Supplementary Figure 5. Experimentally observed nematic patterns of fd-virus rods confined in annular microchambers. The seven patterns were classified as: **a**. D_2, **b**. D_3, **c**. D_∞, **d**. N, **e**. A_1, **f**. A_2 and finally **g**. A_+. **Left column**. schematic. Black lines depict orientation of nematic director. Black points denote positive singularities. White points denote negative singularities. **Center column**. Representative images of the nematic director field. Brightness corresponds to maximum intensity projection of acquired time lapse image series. Color corresponds to the time-averaged orientation $\langle\theta\rangle$ of the nematic director according to the calibration wheel. Scale bars a–f: 5 μm. Scale bars g: 10 μm. **Right column**. Probability of

pattern occurrence as a function of the inner radius (R_{inner} / R_{outer}) and outer radius (R_{outer} / µm) of the microchambers.

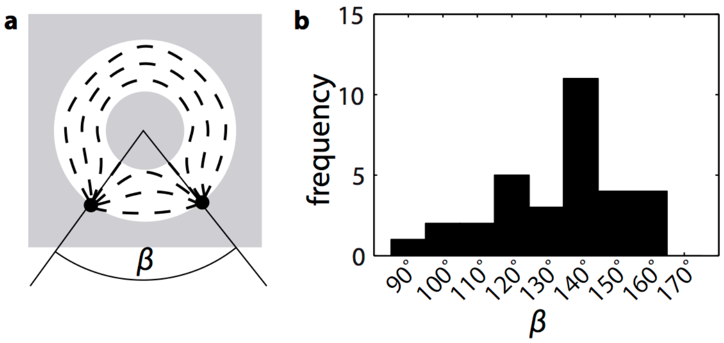

Supplementary Figure 6. Distribution of angles, *β*, between the two +1/2 defects observed in asymmetric A_2 patterns. a. Schematic representing the angle *β* formed by the two point defects and the center of the circle. **b**. Histogram of observed values of *β*.

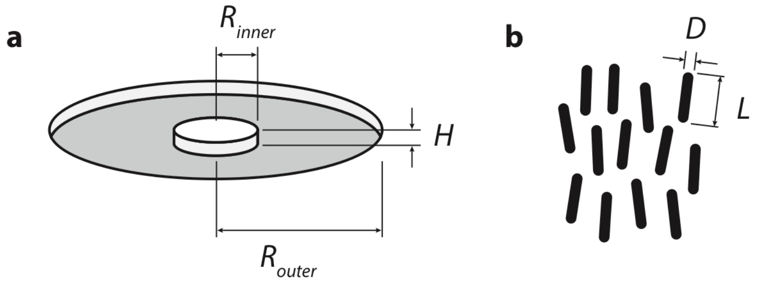

c

	Simulation	Experiment
R_{outer}	$40/D$	5–50 µm
R_{inner}	0–0.5 R_{outer}	0–0.7 R_{outer}
H	1–6 D	1–3 µm
L	15–25 D	133 D
D	1	6.6 nm
	$K_1 < K_3$	$K_1 \approx K_3$

Supplementary Figure 7. Comparison of length scales probed in simulations and experiments. a. Schematic of an annular chamber with outer radius R_{outer}, inner radius R_{inner}, and vertical height *H*. **b**. Schematic of rods with thickness *D* and length *L*. **c**. Table comparing values of R_{outer}, R_{inner}, *H*, *D*, and *L* between simulation and experiment, as well as the relationship between the splay and bend moduli K_1 and K_3.

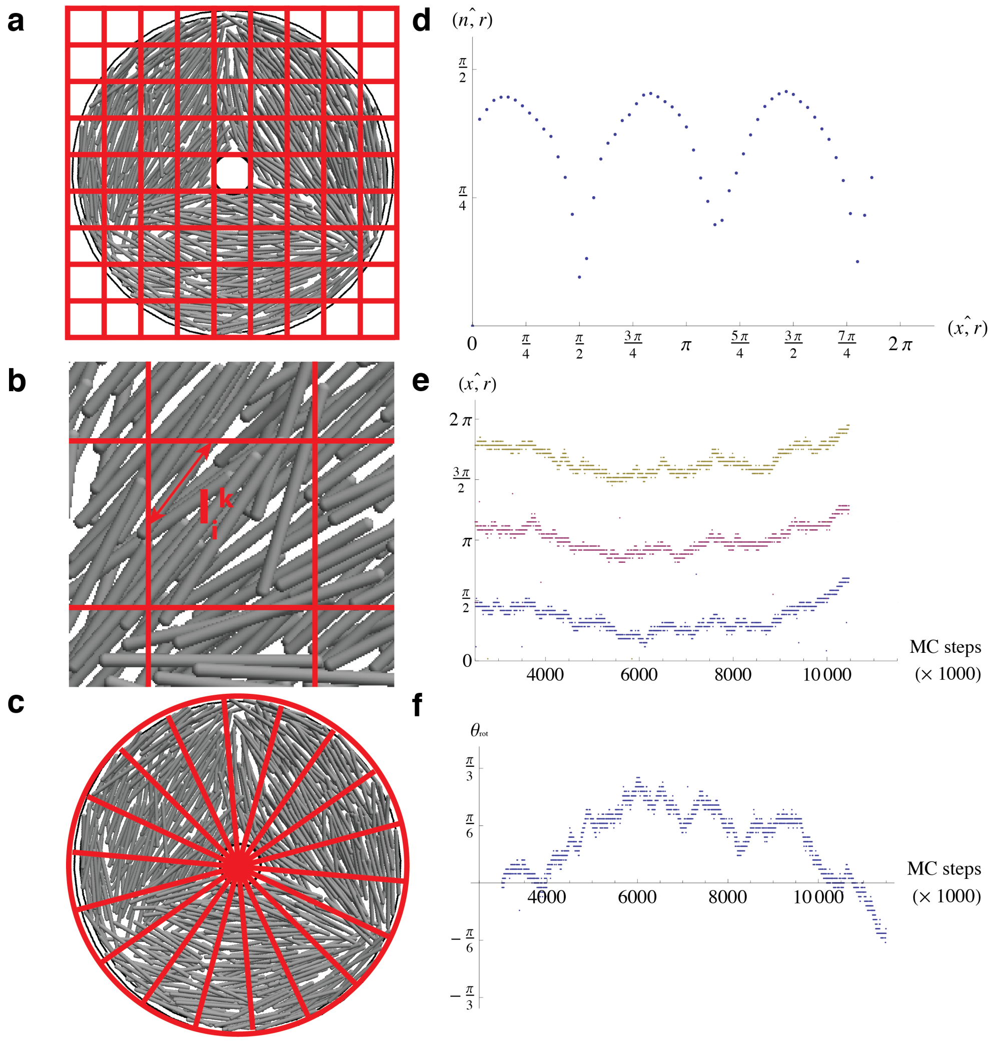

Supplementary Figure 8. **Computation of the local tensor order parameter. a** Top view of the confining container divided into cuboid subvolumes. **b** Top view of a subvolume. **c** Top view of the confining container divided into circular sectors. **d** Minimum angle between average orientation angle of the particles and the radial direction versus the polar angle of the confining container. **e** Location of the three defects, expressed in terms of the polar angle of the container, over the course of the simulation after equilibration. **f** Rotation angle for each configuration.

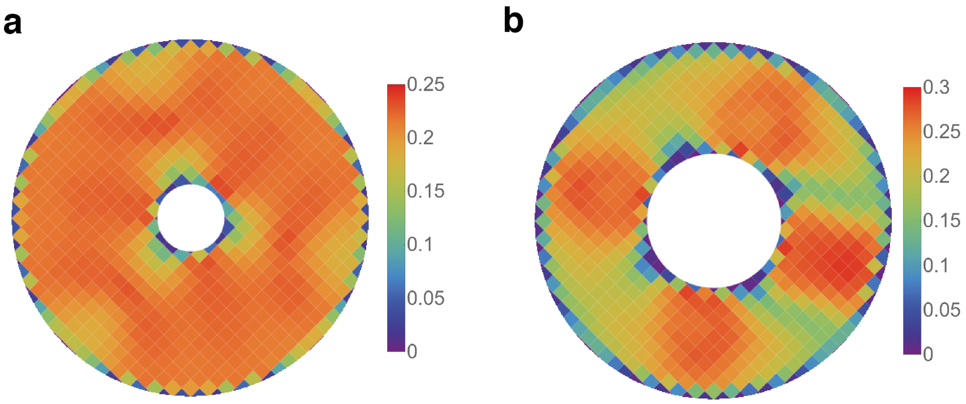

Supplementary Figure 9. Average volume fraction per bin. Images correspond to the data shown in the first and second column of Figure 2 from the main text. For both panels the average packing fraction is $\eta = 0.20$.

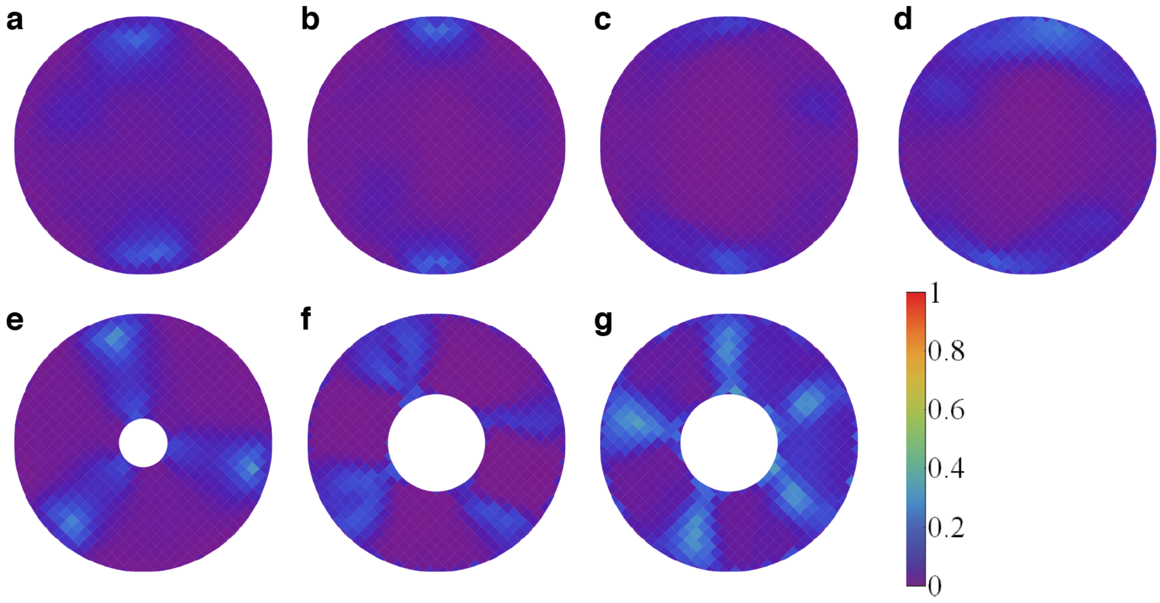

Supplementary Figure 10. Standard deviation of the scalar order parameter. Panels **a-d** correspond to the data showed in Figure 1 from the main text, panels **m** to **p** respectively. Panels **e-g** show the standard deviation for the results presented in Figure 2, panels **g** to **i**, respectively.

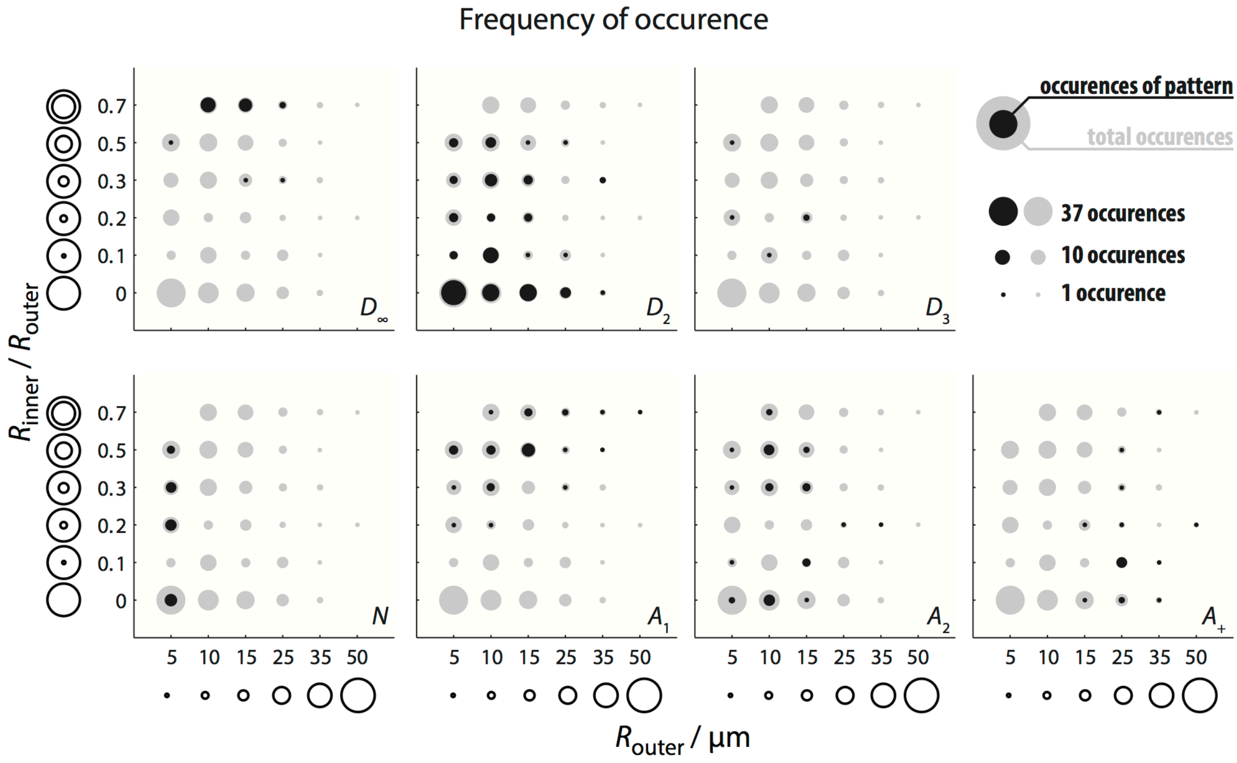

Supplementary Figure 11. **Frequency of experimentally observed pattern occurrence.** Data presented covers all chamber geometries investigated. Black circles denote number of occurrences of a given pattern. Gray circles denote total number of chambers analyzed. Circle area depicts frequency (legend, top-right).

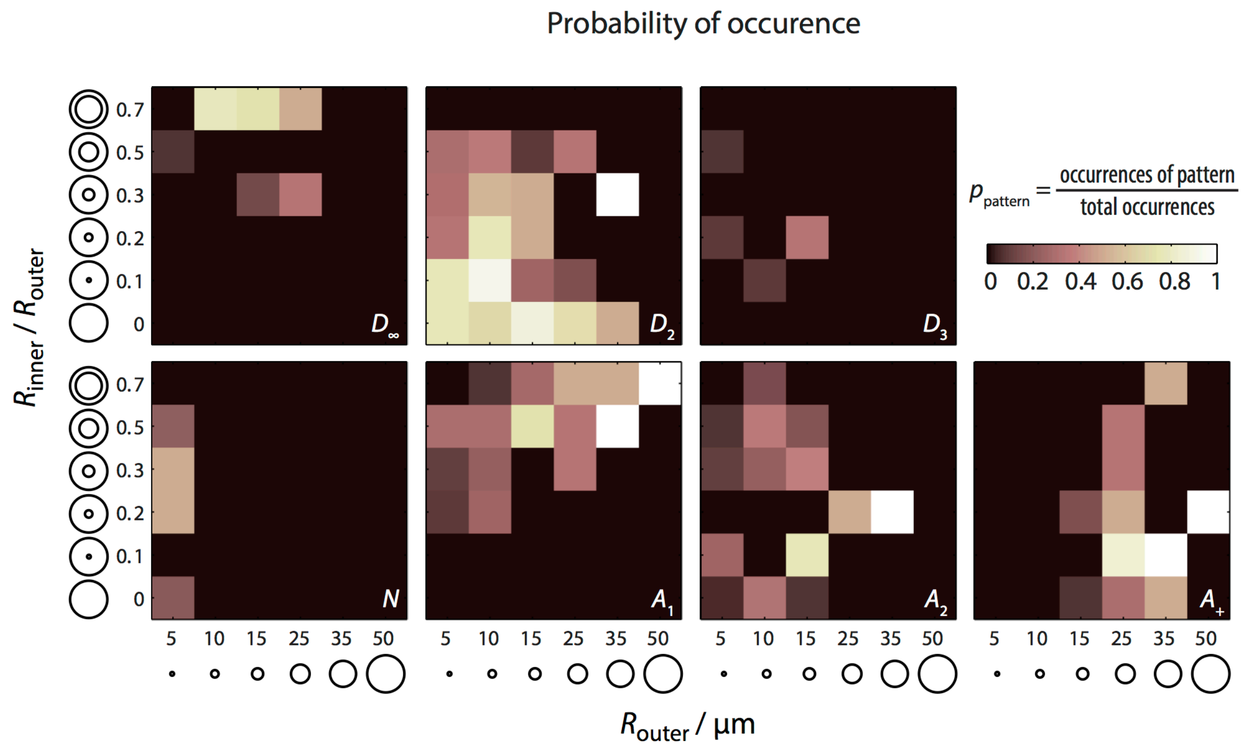

Supplementary Figure 12. Probability of experimentally observed pattern occurrence. Data presented covers all chamber geometries investigated. Color denotes probability (legend, top-right), defined by the number of occurrences observed divided by the total number of chambers analyzed.

Supplementary Table 1. Overview of the defects observed for confinement in a circular geometry

Circular geometry		
**Pattern**	**In-plane defects**	**Vertical defects**
B_i	Two point defects (each with topological charge $+1/2$).	Two line defects.
B_b	Two point defects (each with topological charge $+1/2$).	Two line defects.
B_o	No defects inside the chamber.	No defects.
B_∞	No defects inside the chamber.	No defects.

Supplementary Table 2. Overview of the defects observed for confinement in an annular geometry

Annular geometry		
Pattern	**In- plane defects**	**Vertical defects**
D_2	Two point defects (each with topological charge $+1/2$).	Two line defects.
D_3	Six point defects (three pairs each of them composed of a $+1/2$ and a $-1/2$ topologically charge point defects) and three line defects (connecting the pairs of oppositely charged point defects).	Six line defects and three wall defects (each wall is bordered by two lines).
$D_{n\ (n\geq 4)}$	n line defects.	n wall defects.
D_∞	No defects.	No defects.

Supplementary Note 1
Mean-field calculation

In order to assess the effect of the finite size of the particles on the pattern observed in the simulations and the experiments we perform mean-field calculations, using a microscopic mean-field theory[1] . Our model is designed in such a way as to allow the variation of the full tensor order parameter, accommodating changes in both the scalar order parameter and in the orientation direction. Assuming the system is homogeneous along the z-direction, we formulate our model in two dimensions. The effective free energy functional has the form:

$$\begin{aligned}\beta F[\rho^{(1)}] = &\iint_A \int_{S^1} d\mathbf{r} d\widehat{\omega} \rho^{(1)}(\mathbf{r},\widehat{\omega}) \left\{\log\left(\mathcal{A}_T \rho^{(1)}(\mathbf{r},\widehat{\omega})\right) - 1\right\} \\ &-\frac{J}{2}\iint_A d\mathbf{r} Q_{\mu\nu}(\mathbf{r}) Q_{\mu\nu}(\mathbf{r}) + \frac{L_1}{2}\iint_A d\mathbf{r} \partial_\mu Q_{\nu\tau}(\mathbf{r}) \partial_\mu Q_{\nu\tau}(\mathbf{r}) \\ &+\frac{L_2}{2}\iint_A d\mathbf{r} \partial_\mu Q_{\mu\nu}(\mathbf{r}) \partial_\tau Q_{\tau\nu}(\mathbf{r}) + \frac{L_2}{4}\oint_{\partial A} ds \hat{\mathbf{b}}(\mathbf{r}(s)) \{Q_{\tau\nu}(\mathbf{r}(s)) \partial_\tau Q_{\mu\nu}(\mathbf{r}(s)) \\ &- Q_{\mu\nu}(\mathbf{r}(s)) \partial_\tau Q_{\tau\nu}(\mathbf{r}(s))\} + \frac{W}{2}\oint_{\partial A} ds \left(\hat{\mathbf{b}}(\mathbf{r}(s)) \cdot \hat{\mathbf{n}}(\mathbf{r}(s))\right)^2 ,\end{aligned}$$

where S^1 is the dimensional unit circle, $\rho^{(1)}(\mathbf{r},\widehat{\omega})$ the one-particle areal density distribution, $\widehat{\omega}$ is planar unit orientation vector and $\mathbf{r}$ the position of the particles, $\hat{\mathbf{b}}(\mathbf{r}(s))$ is the outwards normal to the confining wall, $\hat{\mathbf{n}}(\mathbf{r}(s))$is the local director, and

$$\mathbf{Q} = \int_{S^1} \frac{d\mathbf{r} d\widehat{\omega} \rho^{(1)}(\mathbf{r},\widehat{\omega})(2\widehat{\omega}\otimes\widehat{\omega} - \mathbb{I}_2)}{\int_{S^1} d\mathbf{r} d\widehat{\omega} \rho^{(1)}(\mathbf{r},\widehat{\omega})}$$

is the 2D tensor order parameter. The highest eigenvalue of this tensor corresponds to the scalar order parameter (describing the amount of liquid crystalline order in the systems) and its corresponding eigenvector gives the orientation. The coupling constant J and two elastic constants L_1 and L_2 describe the bulk behavior of the system, whereas W controls the wall anchoring strength. The latter parameter has to be chosen independently. We minimize the above effective free energy functional for given values of J, L_1, $L_2$and W, using a simulated annealing Monte Carlo technique [2].

For the disk geometry, we find that all the patters observed in the simulations are reproduced by our field theory (Supplementary Fig. 1). The location of the defects is controlled by the relative strength of the wall coupling constant W relative to the elastic constants $L_1$and L_2. For high wall coupling constants, the defects are located inside the volume (B_I pattern), the wall being coated by a nematic film. For smaller W, the defects relocate to the boundary of the container (B_b pattern) and, upon further decrease of the wall coupling constant, the defects disappear from inside the container, with only a bend in the nematic director being observable (B_O pattern). Finally, for very small wall coupling constants, the liquid crystalline field is not disturbed by the presence of the wall (B_∞ pattern). However, in the annular geometry, we are only able to find, as minimum energy configurations of the system, the bipolar pattern (D_2) and the infinite symmetry pattern D_∞ (appearing for $R_{\text{inner}}/\ R_{\text{outer}} > 0.1$). We occasionally see the experimentally observed A_1 and

A_2 patterns but these have higher energy. The higher fold symmetry patterns (D_3-D_8) do not appear, clearly indicating that these patterns are the result of the finite size of the particle.

Supplementary Note 2
Order parameters

Local order parameter tensor. To globally characterize the order of the system we use the standard three dimensional (3D) second rank tensor order parameter [3]:

$$\mathbf{Q}_3 = \frac{1}{N}\langle\sum_i \frac{1}{2}(3\hat{\boldsymbol{\omega}}_i \otimes \hat{\boldsymbol{\omega}}_i - \mathbb{I}_3)\rangle,$$

where $\hat{\boldsymbol{\omega}}_i$ is a unit vector along the symmetry axis of the rods, $i = 1, \cdots, N$ a label for the particles, and angle brackets denote ensemble averaging. While this global order parameter provides information aspects such as the equilibration of the system, in order to study the orientational patterns of the confined liquid crystal, which change over length scales smaller than the size of the system, we need a spatially resolved version of the above tensor order parameter. We construct this local version by dividing the container in small cuboidal sub-volumes, which have a square cross-section and the same height as the container. The local version of the tensor order parameter reads [4]:

$$\mathbf{Q}_3^k = \frac{1}{\sum_i l_i^k}\langle\sum_i l_i^k \frac{1}{2}(3\hat{\boldsymbol{\omega}}_i \otimes \hat{\boldsymbol{\omega}}_i - \mathbb{I}_3)\rangle,$$

where k labels the subvolumes, $\hat{\mathbf{u}}_i$ and l_i^k is the length of the cylindrical part of particle i contained in the subvolume k. The length weighting ensures that the contribution to the order parameter of each particle is given its proper relative weight. As scalar order parameter we will use the largest (positive) eigenvalue of the tensor, a number in the range 0 to 1, which we denote by S^k. The corresponding normalized eigenvector $\hat{\mathbf{n}}^{\boldsymbol{k}}$ points along the average direction of alignment in subvolume k.

The effect of planarization. The geometry of the systems we consider involves a finite volume bounded by two closely spaced, plane parallel surfaces. We take the normal to these surfaces to be in the z direction of the laboratory frame. We observe planarization, since the particle orientations are only slightly tilted out of the x–y plane, and the particle orientations can thus be denoted by

$$\hat{\boldsymbol{\omega}} = \sqrt{1-\varepsilon_\perp^2}\hat{\boldsymbol{\omega}}_\parallel + \varepsilon_\perp \hat{\mathbf{z}}$$

where $\hat{\boldsymbol{\omega}}_\parallel$ is a unit vector with only x and y components and $\varepsilon_\perp \leq 0.1$, corresponding to the observed out-of-plane tilts of maximally around $\sim 10^\circ$. Inserting this expression into the standard representation of the 3D order parameter tensor yields

$$\mathbf{Q}_3 = \frac{3}{2}\langle(1-\varepsilon_\perp^2)\hat{\boldsymbol{\omega}}_\parallel \otimes \hat{\boldsymbol{\omega}}_\parallel\rangle - \frac{1}{2}(\hat{\mathbf{x}} \otimes \hat{\mathbf{x}} + \hat{\mathbf{y}} \otimes \hat{\mathbf{y}}) + \frac{1}{2}\langle 3\varepsilon_\perp^2 - 1\rangle \hat{\mathbf{z}} \otimes \hat{\mathbf{z}} \equiv \mathbf{Q}_3^\parallel + \mathbf{Q}_3^\perp,$$

where for simplicity's sake we have incorporated the sum over the individual particles and the length weighting into the definition of the averaging implied by the angle brackets. Note that the first two tensors are entirely planar (i.e. only have non-zero components in the indices x and y). Without loss of generality, we can therefore identify them with their projections on the x–y plane. To lowest order in $\varepsilon_\perp$ we then have

$$\mathbf{Q}_3^{\parallel} \sim \frac{3}{2}\langle\hat{\boldsymbol{\omega}}_{\parallel} \otimes \hat{\boldsymbol{\omega}}_{\parallel}\rangle - \frac{1}{2}\mathbb{I}_2$$

$$\mathbf{Q}_3^{\perp} \sim -\frac{1}{2}\hat{\mathbf{z}} \otimes \hat{\mathbf{z}}$$

We now recall that the standard 2D tensor order parameter is defined as [3]:

$$\mathbf{Q}_2 = 2\langle\hat{\boldsymbol{\omega}}_{\parallel} \otimes \hat{\boldsymbol{\omega}}_{\parallel}\rangle - \mathbb{I}_2$$

and so

$$\mathbf{Q}_3^{\parallel} \sim \frac{3}{4}\mathbf{Q}_2 + \frac{1}{2}\mathbb{I}_2$$

This shows that as long as we can neglect out-of-plane tilting, the 2D and 3D order parameter tensors are fully equivalent. More specifically, any eigenvector of $\mathbf{Q}_2$ is also an eigenvector $\mathbf{Q}_3^{\parallel}$, albeit that the eigenvalues are shifted, i.e.

$$\lambda_3^{\parallel} = \frac{3}{4}\lambda_2^{\parallel} + \frac{1}{2}.$$

This shows that in this case the maximal eigenvalue of $\mathbf{Q}_3$ also corresponds to the maximal eigenvalue of $\mathbf{Q}_2$, hence the two order parameters are fully equivalent. This justifies our use of $\mathbf{Q}_2$ in the mean-field calculations described below.

Supplementary Note 3
Characterization of the topological defects

In the circular geometry we observed four types of bipolar patterns, two of which are characterized by defects while two appear to be defect-free (see Supplementary Table 1). All patterns are almost perfectly planar, the organization along the vertical axis being essentially a stack of planar configurations. The B_i pattern is characterized by two defects, each with topological charge $+1/2$, located at a finite distance from the wall; the wall itself is "coated" with a nematic film with director tangent to the wall. The pattern B_b is the standard one observed for the continuum case (see e.g. ref. [5]), with two antipodal $+1/2$ defects located at the boundary. In both patterns, the point defects in the horizontal plane are the endpoints of two lines of defects running along the vertical axis. For the B_o pattern there are no defects present within the simulation volume, but the nematic arrangement is distorted. The field lines are seen to converge towards two points located outside the volume, where they form a pair of virtual defect points similar to the virtual boojums predicted for nematics confined in a spherical geometry (see refs [6,7]). The B_∞ pattern is the limiting case of the B_o pattern, when the two virtual singularities are located infinitely far from the simulation volume, yielding a homogeneously aligned phase in the interior.

The location of the defects can be understood by considering the relative strength of two competing alignment tendencies: the particle-particle alignment and the alignment of the

particles to the wall. This interplay is also fully captured by mean-field calculations (see above), which reproduce all patterns mentioned.

In case the coupling of the liquid crystal to the wall is strong, such that the alignment follows the local direction of the wall, the global organization of the confined system must follow the topological constraints imposed by the confining geometry. In that case the net topological charge of the defects in the liquid crystal should be equal to the Euler characteristic of the enclosing surface (Poincare's theorem, see e.g.[8]). A disk has Euler characteristic $\chi = 1$ and thus the total topological charge must be +1. This can be achieved by two $+1/2$ defects, located either inside the disk as observed for the B_i, or in case of the B_b at the boundary itself. If the density, or equivalently the aspect ratio of the particles, is increased, the interparticle alignment dominates over the wall alignment, and the particles no longer align perfectly to the wall. This is the case for the B_o pattern, where nevertheless the nematic phase appears confined to a virtual elliptical shape, whose Euler characteristic of $\chi = 1$ is being compensated by the two virtual $+1/2$ defect points. Topologically, the B_∞ is the limiting case in which the length of the major axis of this virtual ellipse approaches infinity.

In the annular geometry we observe four types of patterns: a bipolar one (D_2) which is similar to the B_b pattern in the circular geometry, a three-fold symmetric one (D_3), a number of higher symmetry patterns ($D_{n\,(n\geq4)}$) and a defect free pattern D_∞ (see Supplementary Table 2). The D_3 pattern features three pairs of in-plane point singularities connected by radially oriented in-plane defect lines. Each of these defect pairs has a defect with in-plane topological charge $-1/2$ located closest towards the centre and one with charge $+1/2$ closest to the outer wall. Again, these in-plane structures are the endpoints/lines of corresponding lines/walls running vertically through the system. An annulus has Euler characteristic $\chi = 0$ and therefore we expect the sum of topological charge of the nematic defects that it encloses to vanish in case of strong wall alignment, which clearly is the case for the D_3 pattern. The $D_{n\,(n\geq4)}$ patterns exhibit n more complex in-plane radial line defects. A deeper analysis of these structures would be required to determine their significance vis-a-vis the topological constraints. Finally, the defect-free D_∞ pattern trivially satisfies the topological constraints. It is also the lowest free-energy state predicted by continuum theories in the strong anchoring limit (see Supplementary Note 1). However, here the pattern only appears when the distance between the outer and inner walls precludes the particles to have an orientation with a significant component in the radial direction. This again underscores our conclusion that the finite symmetry D_n patterns, observed both in the experiments ($n = 3$) and simulations ($n \geq 3$), are indeed due to finite particle-size effects.

Supplementary Note 4
Image Analysis

We developed an algorithm to quantify the orientation of the nematic director for each image pixel, given a time series (with N frames) of fluorescently labeled tracer rods diffusing in a dense, ordered suspension of unlabeled rods. In short, we determine rod orientations θ for each frame, and average over frames to get the mean orientation $\langle\theta\rangle$ per pixel.

Step 1: Given an image of fluorescent rods (Supplementary Fig. 3a), we compute their orientations θ (Supplementary Fig. 3b) and the gradient energy, which quantifies the contrast between bright and dark pixels (Supplementary Fig. 3c). This step is implemented using OrientationJ, which is a freely-available ImageJ plugin originally developed to track collagen

and elastin fibers [9,10]. This routine computes structure tensors constructed of the spatial gradients of fluorescence intensity around each pixel (x,y). Determining the eigenvectors of a structure tensor yields the characteristic orientation θ of the fluorescence intensity of a small region ($x\pm\sigma$, $y\pm\sigma$) around each pixel (x,y). We set the parameter $\sigma = 3$ px, which corresponds to the typical length scale of a rod. Furthermore, the trace of the structure tensor yields the gradient energy. Note that this quantity should not be confused with a physical energy. Rather, it is related to the notion of signal energy [11].

Step 2: The energy image from Step 1 is thresholded (Supplementary Fig. 3d) using Otsu's method [12]. This yields a binary image comprising connected components of bright pixels (1) against a dark background (0). Bright pixels in this image correspond to points at or near a fluorescently labeled virus particle.

Step 3: The orientation image from Step 1 is masked using the threshold from Step 2 (Supplementary Fig. 3e). The result is a set of orientation measurements θ only for pixels at or near a fluorescently labeled virus particle. We thus discard orientation measurements of background pixels.

Step 4: Steps 1–3 are repeated for the N frames of the dataset (Supplementary Fig. 3f,g). The result is a series of N images produced by Step 3 (Supplementary Fig. 3h,i).

Step 5: The average orientation $<\theta>$ per pixel is determined given the N images from Step 4 (Supplementary Fig. 3j,k). Each pixel can have up to N orientation measurements, depending on how often it passes the threshold from Step 2. Usually, background pixels never pass the threshold from Step 2 and therefore do not have orientation measurements. As shown in Supplementary Fig. 4c, the number of orientation measurements per pixel is typically $n \sim 10^2$, meaning that most areas are well-sampled.

The von Mises distribution. Computing the arithmetic mean is not a suitable method to determine average orientation $<\theta>$ since the orientation θ is a circular quantity, which takes on values over a finite range (between −90° and 90°) that is periodic (−90° = 90°). Computing the arithmetic mean can give incorrect average orientations: we should expect the two measurements −89° and 89° to average out to 90°, but the arithmetic mean yields 0°. In order to accurately determine average orientation $<\theta>$, we first consider the von Mises distribution (a.k.a. circular normal distribution), which is the circular analog of the Gaussian distribution [13]:

$$p(\alpha) = \frac{e^{\kappa\cos(\alpha-\langle\alpha\rangle)}}{2\pi I_0(\kappa)},$$

where α is a circular quantity that varies in the range $[-\pi, \pi)$ and usually corresponds to an angle or phase. The von Mises distribution is parametrized by two parameters: the expectation value $<\alpha>$ and the concentration λ. These two parameters are analogous to the expectation value μ and the inverse of the standard deviation σ^{-1} of a Gaussian distribution. Note that orientation θ varies in the range $[-\pi/2, \pi/2)$, whereas angle α varies in the range $[-\pi, \pi)$. Although most circular quantities are measured by an angle α (wind direction, phase of a wave), some physical quantities are rather measured by an orientation θ (polarization of light, orientation of apolar rods). In order to relate θ to the von Mises distribution, we multiply the orientation measurements by a factor of 2 to recover angles α. The average angle $<\alpha>$ is computed, and then divided by a factor of 2 to recover the average orientation $<\theta>$. The concentration λ remains unchanged when converting between orientation θ and angle α.

In Step 5, we use the "CircStat" MATLAB toolbox[14] to compute $<\theta>$ for each pixel (Supplementary Fig. 4a), which yields the orientation of the nematic director. We also compute the concentration λ of the distribution for each pixel (Supplementary Fig. 4b). In

principle, λ can be used as a measure of the order parameter of the liquid crystal: higher values of λ indicate a more sharply peaked distribution.

Supplementary Note 5
Experimentally observed nematic patterns

In the main text, we have shown experimental evidence for confined nematic patterns of colloidal fd-virus suspensions that exhibit two-, three-, and infinite-fold symmetries. These patterns occur with a probability that depends on chamber size and inner hole radius. In addition to these three patterns, we observe four additional types of patterns. In this section, we describe all seven patterns in detail, as well as their probability of occurrence as a function of chamber shape (R_{inner} / R_{outer}) and chamber size (R_{outer} / μm).

The two-fold symmetric pattern D_2 contains two +½ singularities on opposite ends of the chamber (Supplementary Fig. 5a). It is most probable in disk-shaped chambers without a central hole (R_{inner} / R_{outer} = 0). D_2 patterns also form in chambers with a hole (R_{inner} / R_{outer} > 0), but with a probability that decreases sharply with increasing hole size. In chambers with a central hole, the D_2 patterns exhibit two -½ singularities on the inner wall, which are co-linear with the outer +½ singularities (Supplementary Fig. 5a, middle column, right image). The occurrence of the D_2 pattern also depends on the chamber size, decreasing with increasing R_{outer} and being zero for the largest chambers having an outer radius of 50 μm. The experimentally observed D_2 patterns correspond to the B_b patterns observed in the simulations (cf. main text, Fig. 3b).

The three-fold symmetric pattern D_3 has three evenly spaced +½ singularities at the periphery of the chamber, accompanied by three evenly spaced -½ singularities at the inner wall (Supplementary Fig. 5b). This pattern only occurs for chambers with a small but finite hole size (R_{inner} / R_{outer} = 0.2). Hole diameters for chambers with D_3 patterns vary in the range 2–6 μm, corresponding to ~2.5–7.5 fd-rod lengths. Furthermore, the D_3 pattern occurs in smaller chambers with R_{inner} up to 15 μm. The experimentally observed D_3 patterns correspond to the three-fold symmetric patterns observed in the simulations (cf. main text, Fig. 2a).

The pattern with infinite-fold symmetry D_∞ does not exhibit real singularities, and the rods remain aligned to the boundaries (Supplementary Fig. 5c). This pattern is most likely to occur in narrow ring-shaped chambers with a large hole in the middle, with R_{inner} / R_{outer} = 0.7. Furthermore, this pattern occurs exclusively in the smallest chambers with R_{outer} up to 25 μm.

In the smallest chambers (R_{outer} = 5 μm) we observe nematic liquid crystals where rods align with each other but not along the circular contour of the chamber (Supplementary Fig. 5d). We denote this pattern, which resembles a bulk nematic state, with the symbol *N*. These patterns occur over a broad range of hole sizes, but only when R_{inner} = 5 μm. The experimentally observed *N* patterns correspond to the B_o and B_∞ patterns observed in the simulations (cf. main text, Fig. 3c,d).

In the experiments, we also observe three further patterns with lower symmetries. Some chambers exhibit only one +½ singularity at the outer wall and one -½ singularity at the inner wall (Supplementary Fig. 5e). Because of the lack of non-trivial rotational symmetry, we denote this asymmetric pattern A_1 for "asymmetric, one singularity". This pattern was mostly observed in thin, annular chambers (R_{inner} / R_{outer} = 0.5 and 0.7). The probability of finding A_1 increases somewhat with increasing chamber size, but the pattern is observed over the entire range of chamber sizes (5-50 μm). We also observe another asymmetric pattern that exhibits

two pairs of +½ and -½ singularities, similar to D_2. But the defects are not positioned at polar opposites (Supplementary Fig. 5f). We call this pattern A_2 ("asymmetric, two singularities"). This pattern occurs over a wide range of hole and chamber sizes with no particular preference. We quantify the relative positions of the two singularities for A_2 patterns by the angle β (Supplementary Fig. 6a), and find a broad angle distribution ranging from 90 to 160°, with a peak at $\beta = 140°$ (Supplementary Fig. 6b). (Note that this distribution excludes chambers that have been classified as D_2 and therefore exhibit $\beta = 180°$.) In some cases, multiple singularities are scattered across the interior of the chamber, and the nematic director field exhibits chaotic-looking patterns (Supplementary Fig. 5g). We denote such structures with the symbol A_+ ("asymmetric, 3 or more singularities"). We observed this pattern mostly in large chambers with small holes (R_{inner} / R_{outer} = 0.1 and R_{outer} > 25 μm).

Supplementary Note 6
Comparison of experiment and simulations

The D_3 pattern was observed over a narrow range of R_{inner}, both in simulation and experiment. However, the ranges of R_{inner} do not coincide. In the experiments, the D_3 pattern occurred for R_{inner} / L = 1.2–3.7. These values are greater than expected from the relation between R_{inner} and L (cf. main text), where we would expect R_{inner} / $L \sim 12^{-1/2} = 0.29$ (cf. main text, Fig. 3, vertical gray dashed line corresponding to R_{inner} = 4.35 D). This relation agrees well with simulations performed with the shallowest chambers (H / D = 1), where D_3 was found in the range R_{inner} / L = 0.2–0.5 (or R_{inner} / D = 3–7.5). We suspect that these different ranges of R_{inner} arise from the fact that experiment and simulation could not be performed with identical parameters (Supplementary Fig. 7). In particular, two effects may dominate. First, the confinement in the z-direction (height H) was less stringent in experiments (H = 1–3 μm, H / L = 1.1–3.4, H / D = 146–452) than in simulations (H / D = 1–6). The simulations demonstrated that higher chambers (larger H) exhibit lower-order symmetries (cf. main text, Fig. 3). In particular, the range of R_{inner} where D_3 occurs increases by a factor of two as H / D increases from 1 to 6, resulting in R_{inner} / L = 0.27–1. This range does not overlap with the experimentally observed range of R_{inner} / L = 1.2–3.7, but does come close. We expect that simulations in thicker chambers would recover better agreement with experiment. Second, the rods used in simulation are rigid, likely resulting in $K_1 < K_3$, where K_1 is the Franck elastic constant for splay and K_3 for bend strains. Meanwhile fd viruses are semiflexible, with a persistence length of 2.2 μm [15]. As a result, $K_1 \sim K_3$ [16]. It is possible that the differences in Frank elastic constant between experiment and simulation can account for the different ranges in R_{inner} where D_3 occurs.

Experiment and simulation agree on the occurrence of N patterns. In the experiments, we found N patterns only for the smallest chambers investigated (R_{outer} = 5 μm, R_{outer} / L = 5.7). In the simulations, the N pattern occurred when the rod length L increased such that R_{outer} / L = 2 or 1.6. (cf. main text, Fig. 1). Although the experimental and simulated values of R_{outer} / L do not coincide, it is likely that this mismatch can again be accounted for by differences in chamber height and/or Frank elastic constants.

One important question is whether the experimentally observed patterns represent a thermodynamic equilibrium state. Although we cannot strictly rule out the possibility of nonequilibrium behavior, the nematic patterns we observed were stable over the course of 1–24 h after sample assembly and were reproducible over a large number of chambers. Interestingly, the four experimental patterns that appear as equilibrium states in simulations

(D_2, D_3, D_∞, and N) were most likely to occur in small chambers (R_{outer} ≤ utere mos Supplementary Fig. 11a1g.lementary kely to occur in sA_1, A_2, and A_+ patterns tend to occur in large chambers (R_{outer} ≥ 25 rerns teSupplementary Fig. 11e1g.lementary to occur in large chambers (rs (tally observed patterns represent a thermodynamic equilibrium state. Although we cannot strictly rule out the possi

Supplementary Note 7
Quantification of defects

To characterize the defects, we use an angular defect parameter δ measuring the variation in the direction of alignment around a point. This parameter is defined as:

$$\delta = \min\angle(\hat{\mathbf{n}}^1, \hat{\mathbf{n}}^2) + \min\angle(\hat{\mathbf{n}}^3, \hat{\mathbf{n}}^4),$$

where $\hat{\boldsymbol{n}}^j$ are orientations of neighboring sub volumes (see Supplementary Fig. 8b).
Due to the high symmetry of the confining containers we consider, there is no preimposed preferential direction of alignment. For example, in the case of a cylindrical box, all bipolar configurations are equivalent, regardless of the angle that the line defined by the two defects makes with the x-axis. In the course of a simulation the pattern therefore rotates around the symmetry axis of the confining box. We compute the scalar order parameter and the average orientation not on the basis of a single configuration, but as an average of independent configurations sampled throughout the simulation time. For an ergodic system in thermal equilibrium this type of ensemble averaging is equivalent to time averaging. If we average over these configurations without accounting for the location of the defects, the patterns will be washed out. Averaging, for example, over bipolar configurations with the pair of antipodal defects homogeneously distributed around the box results in an overall isotropic-like configuration. To prevent this problem we developed a strategy for locating the defects and then rotating the configurations in such a way that the defects are located always at the same spot. Due to the rotational symmetry of the confining container around its center line, both in the disk and the annular geometry, we expect the defect structures, if any, to be located radially. Therefore it is convenient to divide the container into circular sectors (see Supplementary Fig. 8c). In each of these sectors we compute the average orientation of the particles $\mathbf{n}$. The minimum angle between the average orientation and the radial unit vector to the center of the circular sector will run from 0 to π/2, with 0 corresponding to particles arranged radially and π/2 to particles aligned to the wall. A sharp drop in this minimum angle with respect to the polar angle of the confining container indicates the presence of a defect structure.
As an example, for the 3-fold structure that we see by eye in the configuration shown in Supplementary Fig. 8 a and c, we observe, in panel d of the same figure, 3 sharp minima corresponding to the angles where the nematic-like domains meet. Extracting these local minima for each configuration, allows us to monitor the location of these defects over the course of the simulation (see Supplementary Fig. 8e). The defect structures move synchronously, the pattern rotating as a whole, in both directions, around the symmetry axis of the container. By taking a single configuration as reference (for example the first one after the system is considered equilibrated) we compute the angle θ_m by which we need to rotate the other configurations we use in the averaging in order to obtain equivalent configurations, with the defects overlapping. This rotation angle is plotted in Supplementary Fig. 8f as

function of the simulation time (expressed in Monte Carlo steps). After rotating the configurations we perform the averaging by using the division into cuboid subvolumes. We do not compute the tensor order parameter in the circular sector based subvolumes, but prefer the cuboid one for the final analysis, because in the circular sectors we have no way of differentiating a point defect from a disinclination wall.

Supplementary Note 8
Error estimation for the simulation results

The simulation results shown in Fig. 1 and 2 in the main text are obtained by averaging the local tensor order parameter over 1000-3000 independent configurations sampled throughout the course of a simulation. Only configurations obtained after the system had equilibrated, which we monitor using the global scalar order parameter, are used for the averaging. The local scalar order parameter and orientation, shown in the Fig. 1 and 2 in the main text, are computed as the highest positive eigenvalue and its corresponding eigenvector of the local tensor order parameter (see the subsection Supplementary Note 2 above). In order to estimate the standard deviation of these quantities, we compute, using the same procedure, the desired values by averaging over smaller subsets of configurations (typically 100), and using these subsamples as independent measurements. The results for the scalar order parameter are shown in Supplementary Fig. 10. Within the nematic-like domains the standard deviation is very low and only increases in the vicinity of the defect structures. The increase near defects is due to the fact that the relative position of the defects is not completely fixed: For the disk geometry, for example, the two defect points are not permanently diametrically opposite within the container and in the annular geometry the angle between the two line defects is not exactly 360°/*n*. Another source of error comes from the rotation we perform on the configurations in our averaging procedure, since the rotation angle is binned. For panel d of Supplementary Fig. 10, which corresponds to the B_{∞} infinity pattern, the higher error at the poles corresponds to particles getting trapped between the wall and the nematic-like domain.

We note that the errors in the vicinity of the defect points are not caused by sampling errors due to small numbers of particles, as the defects do not correspond to empty cuboids (see Supplementary Fig. 9). The volume fraction of the cuboids containing the defect structure is, however, lower than the average volume fraction due to the fact that in our averaging procedure we only account for the central line of each spherocylindrical particle, neglecting the spherical caps. In the vicinity of the defects, a higher volume fraction is occupied by the spherical caps of the particles than elsewhere. For the annular geometry, in the region of point defect, the volume fraction is 10% lower than the average density (see Supplementary Fig. 9a). A similar decrease is also observed in the vicinity of the point defects in disk geometry. Close to the wall defects the density is ~20% lower, while inside the nematic-like domains it increases (see Supplementary Fig. 9b). The only cuboids that are almost empty are the ones next to the walls, which very often are partially outside our simulation volume.

Supplementary References

1. Lewis, A. H. *et al.* Colloidal liquid crystals in rectangular confinement: theory and experiment. *Soft Matter* **10,** 7865–73 (2014).
2. Ingber, L. Adaptive simulated annealing (ASA): Lessons learned. *Control Cybern.* **25,** 33–54 (1996).
3. de Gennes, P. G. & Prost, J. *The Physics of Liquid Crystals (International Series of Monographs on Physics).* (Oxford University Press, USA, 1995).
4. Gârlea, I. C. & Mulder, B. M. Defect structures mediate the isotropic-nematic transition in strongly confined liquid crystals. *Soft Matter* **11,** 608–14 (2015).
5. van Bijnen, R. M. W., Otten, R. H. J. & van der Schoot, P. Texture and shape of two-dimensional domains of nematic liquid crystals. *Phys. Rev. E. Stat. Nonlin. Soft Matter Phys.* **86,** 051703 (2012).
6. Prinsen, P. & van der Schoot, P. Shape and director-field transformation of tactoids. *Phys. Rev. E* **68,** 021701 (2003).
7. Prinsen, P. & van der Schoot, P. Continuous director-field transformation of nematic tactoids. *Eur. Phys. J. E. Soft Matter* **13,** 35–41 (2004).
8. Kinsey, L. C. *Topology of Surfaces.* (Springer Science & Business Media, 1993).
9. Fonck, E. *et al.* Effect of Aging on Elastin Functionality in Human Cerebral Arteries. *Stroke* **40,** 2552–2556 (2009).
10. Rezakhaniha, R. *et al.* Experimental investigation of collagen waviness and orientation in the arterial adventitia using confocal laser scanning microscopy. *Biomech. Model. Mechanobiol.* **11,** 461–473 (2012).
11. Mitra, S. K. & Kaiser, J. F. *Handbook for Digital Signal Processing.* (John Wiley {&} Sons, Inc., 1993).
12. Otsu, N. A threshold selection method from gray-level histograms. *IEEE Trans. Syst. Man, Cybern. Syst.* **9,** 62–66 (1975).
13. Jammalamadaka, S. R. & Sengupta, A. *Topics in Circular Statistics.* (World Scientific Publishing Company, 2001).
14. Berens, P. CircStat: a MATLAB toolbox for circular statistics. *J. Stat. Softw.* **31,** 1–21 (2009).
15. Dogic, Z. & Fraden, S. in *Soft Matter, Vol. 2: Complex Colloidal Suspensions* (eds. Gompper, G. & Schick, M.) 1–86 (Wiley-VCH Verlag GmbH & Co. KGaA, 2005).
16. Dammone, O. *et al.* Confinement Induced Splay-to-Bend Transition of Colloidal Rods. *Phys. Rev. Lett.* **109,** 108303 (2012).
